# Impact of individual actions on the collective response of social systems

**DOI:** 10.1038/s41598-020-69005-y

**Published:** 2020-07-22

**Authors:** Samuel Martin-Gutierrez, Juan C. Losada, Rosa M. Benito

**Affiliations:** Grupo de Sistemas Complejos, Escuela Técnica Superior de Ingeniería Agronómica, Alimentaria y de Biosistemas, Universidad Politécnica de Madrid, Av. Puerta de Hierro, 2, 28040 Madrid, Spain

**Keywords:** Complex networks, Scientific data, Statistics, Applied mathematics

## Abstract

In a social system individual actions have the potential to trigger spontaneous collective reactions. The way and extent to which the activity (number of actions—*A*) of an individual causes or is connected to the response (number of reactions—*R*) of the system is still an open question. We measure the relationship between activity and response with the distribution of efficiency, a metric defined as $\eta =R/A$. Generalizing previous results, we show that the efficiency distribution presents a universal structure in three systems of different nature: Twitter, Wikipedia and the scientific citations network. To understand this phenomenon, we develop a theoretical framework composed of three minimal statistical models that contemplate different levels of dependence between *A* and *R*. The models not only are able to reproduce the empirical activity-response data but also can serve as baselines or null models for more elaborated and domain-specific approaches.

## Introduction

Due to humans’ social nature, the actions of individuals hold the potential to trigger spontaneous collective reactions, leading to complex dynamics. In order to understand human collective behavior, it is necessary to find the laws that relate the individual actions to the collective response of social systems.

This topic has received considerable attention and has been approached from several perspectives^1–4^. From diffusion on networked systems, a field which studies the spread of diseases or information and the emergence of cascading phenomena^5–7^ to *virality*, a property of certain pieces of information that generate a wide response in social systems^8–10^. Other works focus on the Influence Maximization problem, taking advantage of the diffusion mechanisms to find a set of individuals that maximize the response^11–13^. Alternatively, the field of control theory aims to steer the collective behavior of a system by controlling the activity of a few individuals^14, 15^.

Our goal in this work is to develop a theoretical framework that relates the number of actions performed by an actor (an agent or individual) embedded in a social system; that is, her activity (*A*), and the number of reactions that these actions trigger in her peers, or response (*R*). To relate these two magnitudes we generalize the efficiency metric ($\eta = \frac{R}{A}$), introduced by Morales et al. in the context of Twitter^16^, to other social systems.

We follow a well established modeling approach in social physics: explain the macroscopic properties of the system assuming the simplest microscopic interactions between the actors to extract the most fundamental laws^17–23^. The macroscopic property in which we focus is the distribution of efficiency. We have used this metric to analyze three kinds of social systems of different nature: social networks, collaborative networks and citations networks. In particular, we have worked with 14 Twitter conversations around different issues in Spain, Turkey, Palestine, Argentina and Colombia, the editions of the English Wikipedia and the scientific citations data of authors from 14 different countries extracted from the Web of Science.

In Twitter, the activity is the number of original messages posted by a user and the response of the system is the number of retweets received by that user. Another magnitude used in our analysis is the response to single actions (*r*). In Twitter *r* would be the number of retweets obtained by a single tweet. In the scientific citations network, *A* is the number of publications of an author and *R* the number of citations obtained. The variable *r* in this case is the number of citations obtained by one paper. In the context of the Wikipedia collaboration network, we consider *A* as the aggregated number of editions performed by a particular user in any Wikipedia page. The corresponding *R* is the number of editions made by other users in her personal user page. These editions can be considered as messages directed to that particular user. In this case there is no data for the response to a single edition. Therefore, we have defined *r* as the number of editions made on the pages of users whose activity is $A = 1$.

We have found that the efficiency distribution in these three systems has a universal structure with small differences between the datasets, which may indicate the existence of a general mechanism governing the $A-R$ relationship. To reveal that mechanism we have developed three domain-independent minimal statistical models. Taking a parsimonious approach, we start from the most naive model and progressively consider more sophisticated theories with increasingly complex levels of dependence between *R* and *A*. The models are the Independent Variables model (InV), the Identical Actors model (IdA) and the Distinguishable Actors model (DiA). In the InV model the response of the system is independent with respect to the activity of the individual. In the IdA model, the response of the system depends on the activity of the individual, but the system is agnostic with respect to the individual that stimulates it. Finally, in the DiA model the response is determined not only by the activity of the individual, but also by her features. The models are general because no assumption is made about the particular characteristics of the system or its components.

## Results

### Distribution of efficiency

The efficiency metric is defined as the quotient between collective response *R* and individual activity *A*:

1 $$\begin{aligned} \eta = \frac{R}{A} \end{aligned}$$

It can be considered as a proxy for how efficient an individual is at triggering reactions in her peers or as a measure of the system’s inertia to react to the stimuli of the individual. The higher the individual’s efficiency, the lower the system’s inertia.

**Figure 1:**
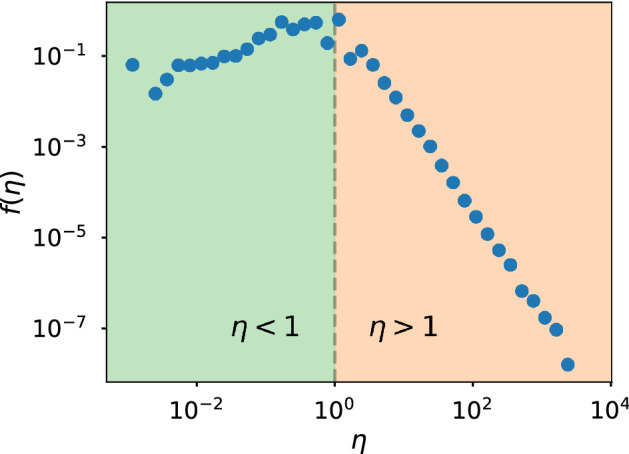
Example of efficiency distribution where the two distinct behaviors that are manifested to each side of the point $\eta =1$ can be appreciated. The data corresponds to the Twitter conversation around the 2015 Spanish General Elections.

Our work is focused on the efficiency distribution, an example of which is presented in Fig. 1. It is characterized by a concave shape with two distinct tendencies for $\eta <1$ (an individual gets less than one reaction per action) and $\eta >1$ (an individual triggers more than one reaction per action). In the work by Morales et al.^16^, they used the Independent Cascade (IC) model on the Twitter follower network to reproduce the empirical distribution of user efficiency and showed that the shape of the distribution was universal for Twitter conversations. However, several questions were left open and some of the empirical results lacked a comprehensive explanation. In particular, they reported evidence for the independence of the efficiency distribution with respect to the functional form of the activity distribution and, from that, conjectured that communication patterns are not dependent on the way users post original messages; that is, that collective response is independent of individual activity.

In this work we go one step further and present evidence for the universality of the structure of the efficiency distribution in two other social systems. We also present the three aforementioned statistical models to provide a comprehensive description of the nature of the efficiency distribution and show the extent to which the activity of the individuals and their particular features influence the response of the system.

### Description of the models

We have calculated the theoretical distributions of efficiency with three different methodologies: Monte-Carlo (MC) simulation, direct computation with discrete probability distributions and derivation of an analytical expression.

Once the basic mechanism of the model is laid out, MC simulation allows a direct implementation of the model’s assumptions. Thus, we use it to compare model and empirical data as well as to verify the results of the other methodologies.

To directly compute the efficiency distribution with the discrete joint probability distribution *p*(*R*, *A*) we follow the method described in the “Methods” section [Eqs. (18) and (19)]. The resulting efficiency distribution is asymptotically exact in the sense that, since the support for the distributions of *A* and *R* is $\mathbb {N}$, an infinite number of terms would be required to actually obtain exact results, but larger values of *A* and *R* have increasingly smaller probabilities, carrying progressively lower weight on the computation and enabling the results to converge for a finite number of terms.

The analytical calculation of the efficiency distribution has been carried out for the InV and IdA models by considering *A* and *R* as continuous random variables. Taking into account the definition of efficiency given by (1) we derive an expression for the probability density function (PDF) of efficiency using the joint probability distribution $\varphi (R,A) = \varphi (\eta A,A)$ (see Section 2 of the Supplementary Information):

2 $$\begin{aligned} f(\eta )={\left\{ \begin{array}{ll} \int _{R_m/\eta }^{\infty } \varphi (\eta A,A) A dA \quad \text {if} \quad \eta \le \frac{R_m}{A_m}\\ \int _{A_m}^{\infty } \varphi (\eta A, A) A dA \quad \text {if} \quad \eta > \frac{R_m}{A_m}\\ \end{array}\right. } \end{aligned}$$

where $A_m, R_m>0$ are the minimum values of *A* and *R*. In our case, $A_m = R_m = 1$ for every dataset. It is worth noting that the two branches of $f(\eta )$ in Eq. (2) correspond to the two characteristic tails of the efficiency distribution.

#### Independent variables model

In the InV model *A* and *R* are considered independent variables with probability distributions *p*(*A*) and *p*(*R*).

A Monte-Carlo simulation can be computed as follows: In a system with *N* individuals indexed by $i=1,2,\ldots , N$, store the empirical data of activity and response in two vectors $\vec {A}$ and $\vec {R}$ such that component *i* of vector $\vec {A}$ corresponds to the same individual as component *i* of vector $\vec {R}$. Next, shuffle each of them independently, such that the correlations that may have been present when each couple $(A_i,R_i)$ corresponded to the same individual vanish. The randomized versions of the vectors, $\vec {A}_{rnd}$ and $\vec {R}_{rnd}$, hold the same values as the originals but with the order of the elements randomly altered. Finally, the efficiency vector $\vec {\eta }_{rnd} = \vec {R}_{rnd} / \vec {A}_{rnd}$ is used to compute the efficiency distribution according to the InV model.

Since *A* and *R* are considered independent, their discrete joint probability distribution is $p(R,A)=p(R)p(A)$. The PDF of efficiency can be obtained by plugging this expression in (18) and (19) of “Methods”. However, for this model we have left out the results of the discrete methodology because we have derived an exact analytical expression.

For the analytical computation of the InV model we consider *A* and *R* as continuous variables with PDFs $f_A(A)$ and $f_R(R)$. Their joint probability distribution can be written as $\varphi (R,A)=f_A(A)f_R(R)$. Plugging this in (2) we obtain:

3 $$\begin{aligned} f^{InV}(\eta )={\left\{ \begin{array}{ll} \int _{R_m/\eta }^{\infty } f_R(\eta A)f_A(A) A dA \quad \text {if} \quad \eta \le \frac{R_m}{A_m}\\ \int _{A_m}^{\infty } f_R(\eta A)f_A(A) A dA \quad \text {if} \quad \eta > \frac{R_m}{A_m}\\ \end{array}\right. } \end{aligned}$$

This expression provides an explanation for a key result presented in^16^, where Morales et al. show that the right tail of the efficiency distribution remains unaltered when the activity distribution is modified. To reach that result, let us assume that $f_R(R)\propto R^{-\gamma _R}$. This power law distribution was used in^16^ as well as in other works to model the distribution of retweets^24^, scientific citations^25^ and incoming editions in Wikipedia^3^. Then, the right tail ($\eta > \frac{R_m}{A_m}$) of the PDF shown in (3) can be written as:

4 $$\begin{aligned} f^{InV}(\eta ) \propto \eta ^{-\gamma _R} \int _{A_m}^{\infty } A^{1-\gamma _R}f_A(A) dA = E_A[A^{1-\gamma _R}] \eta ^{-\gamma _R} \Rightarrow f^{InV}(\eta ) \propto f_R(\eta ) \end{aligned}$$

where $E_A[\cdot ]$ is the expected value with respect to the activity distribution. Therefore, when $f_R(R)\propto R^{-\gamma _R}$, the right tail of the efficiency distribution is proportional to $\eta ^{-\gamma _R}$. That is, in addition to being independent of the activity distribution, its shape is completely determined by the exponent of the response distribution.

To apply the analytical computation of the efficiency distribution for the InV model to empirical data we have fit the empirical distributions of *A* and *R* to a power law with exponential cutoff (or truncated power law) using the powerlaw python module^26^. The functional form of this distribution is the following:

5 $$\begin{aligned} f(x) = \frac{\lambda ^{1-\alpha }}{\Gamma (1-\alpha ,\lambda x_{min})} x^{-\alpha }e^{-\lambda x} \end{aligned}$$

where $\Gamma (s, x)$ is the upper incomplete gamma function. The resulting fits for $f_A(A)$ and $f_R(R)$ for every dataset are presented in the Supplementary Information (SI). When the PDFs of activity and response are power laws with exponential cutoff, the PDF of efficiency adopts the following form:

6 $$\begin{aligned} f^{InV}(\eta )={\left\{ \begin{array}{ll} g(\eta ) \Gamma (2-\alpha _{R}-\alpha _{A},(\lambda _{R} \eta +\lambda _{A})\frac{R_m}{\eta }) \quad \text {if} \quad \eta \le \frac{R_m}{A_m}\\ g(\eta ) \Gamma (2-\alpha _{R}-\alpha _{A},(\lambda _{R} \eta +\lambda _{A})A_m) \quad \text {if} \quad \eta > \frac{R_m}{A_m}\\ \end{array}\right. } \end{aligned}$$

With

7 $$\begin{aligned} g(\eta ) = C (\lambda _{R}\eta +\lambda _{A})^{(\alpha _R+\alpha _A-2)}\eta ^{-\alpha _R} \end{aligned}$$

and

8 $$\begin{aligned} C = \frac{\lambda _R^{1-\alpha _R}}{\Gamma (1-\alpha _R,\lambda _RR_m)} \frac{\lambda _A^{1-\alpha _A}}{\Gamma (1-\alpha _A,\lambda _AA_m)} \end{aligned}$$

#### Identical actors model

A natural extension to the InV model is to consider that the response of the system depends on the activity of the individual. To carry out this extension in a parsimonious way, we realize that the stimuli to which the system reacts occur in a discrete fashion, so we can assume that it reacts to each action (a tweet, a scientific publication, an edition on Wikipedia, etc.) individually, as if they were isolated events. Then, while in the InV model the *aggregate* response of the system was independent of the aggregate activity of the actor, in the IdA model the *partial* response of the system to each single action is independent of the actor. But, as the aggregate response of the system to the activity of an individual is the sum of the partial responses to each of her *A* actions, a dependence between *R* and *A* is induced.

To formalize this idea we introduce the new variable *r* as the response of the system to a single action by any individual. This random variable follows the same distribution *p*(*r*) for all the actors. The aggregate response *R* associated to an actor that performed *A* actions and triggered partial responses $\{r_1,r_2,\dots ,r_A\}$ is $R=\sum _{j=1}^A r_j$. The dependence of *R* on *A* resides on the number of terms of this sum.

To perform a Monte-Carlo simulation of the IdA model, we first fit the *p*(*r*) with the hybrid methodology detailed in the SI and *p*(*A*) to a discrete truncated power law (see the SI for the results). Then, we generate a set of individuals whose activity is assigned according to *p*(*A*). The responses for each of the *A* actions of an individual is randomly generated with *p*(*r*) and then aggregated to obtain her *R*. The efficiency according to this model is directly computed from the (*R*, *A*) tuple associated to each actor.

To get the efficiency distribution of the IdA model from the discrete *p*(*R*, *A*) distribution, we start with the conditional discrete probability distribution of *R* given an activity *A*, which is computed as the $A-fold$ discrete convolution of *p*(*r*) with itself:

9 $$\begin{aligned} p(R|A) = p(r_1)*p(r_2)*\cdots *p(r_A) = p(r)*p(r)*\cdots *p(r) = p^{*A}(r) \end{aligned}$$

Then, the joint probability distribution can be obtained as:

10 $$\begin{aligned} p(R,A) = p(R|A)p(A) = p^{*A}(r) p(A) \end{aligned}$$

The efficiency PDF is obtained by plugging (10) in (18) and (19). The *p*(*r*) and *p*(*A*) distributions used in this methodology are the same as those used in the Monte-Carlo simulations.

To carry out the previous computations with infinite precision we would need an infinite number of values for the *p*(*r*), *p*(*A*) and *p*(*R*, *A*) distributions. To be able to perform the numerical computations, we have used distributions that are bounded at a certain value and we have verified that further increasing the number of values employed do not affect the results. The cut-off values used for the three systems considered are shown in Table 1.

**Table 1:** Cut-off values used to perform the numerical computations for the IdA model.

System	$r_{max}$	$A_{max}$	$R_{max}$
Twitter	$10^6$	$3\times 10^4$	$5\times 10^4$
Wikipedia	$3\times 10^5$	$3\times 10^4$	$5\times 10^4$
Citations	$3\times 10^5$	$2\times 10^4$	$3\times 10^4$

An analytical expression for the efficiency distribution of the IdA model can be derived when *p*(*r*) is modeled as a power law ($p(r)\propto r^{-\gamma _r}$). For this approximation, the activity distribution *p*(*A*) has been modeled as a power law with exponent $\gamma _{A}$, a usual approach in the literature^3, 24^. The corresponding fits are shown in the SI and the resulting expression for the PDF of efficiency is:11where $E_n(\cdot )$ is the generalized exponential integral,  the lower incomplete gamma function and *C* the following normalization constant:

12 $$\begin{aligned} C = \frac{(\gamma _r-1)(\gamma _A-1)}{1+(1-\gamma _A)\Gamma (1-\gamma _A,1)} \end{aligned}$$

#### Distinguishable actors model

In the DiA model the actors are distinguishable, meaning that the system is sensitive to the individual who makes the action and reacts in a different manner depending on her particular features.

This idea can be formalized by considering that the probability distribution of response to single actions depends on the features of the individual that performs the action, summarized in a vector $\vec {s}$. The distribution of aggregate response *R* of the system is computed as the *A*-fold convolution of the $p(r|\vec {s})$ distribution with itself:

13 $$\begin{aligned} p(R|A,\vec {s}) = p^{*A}(r|\vec {s}) \end{aligned}$$

If $\{s_1,s_2,\dots ,s_N\}$ are the components of the feature vector (assume the features are independent discrete variables), the discrete joint probability distribution *p*(*R*, *A*) is obtained as follows:

14 $$\begin{aligned} p(R,A) = \sum _{s_1} \cdots \sum _{s_N} p^{*A}(r|\vec {s}) p(A) p(\vec {s}) \end{aligned}$$

Finally, *p*(*R*, *A*) can be used to compute the efficiency distribution with (18) and (19).

A key point is to find the conditional probability distribution $p(r|\vec {s})$ that characterizes the relationship between the features $\vec {s}$ of the individual and the response *r* of the system to her actions. Unfortunately, this task is not trivial in most cases. In the case of the citation network the literature shows that there are many and varied factors that determine the citation counts of publications^27^, from the quality of the manuscript, to the field of research, the cited references or the reputation of the authors and their institutions. With respect to Wikipedia, some factors that could determine the response to a user could be the topics she is more active on, the age of her user account or her main role (some users may be focused on editing articles, others on moderating discussion pages, etc.).

Among the systems under study we have focused on Twitter, where we have chosen the number of followers *F* of a user as a proxy of her ability to trigger a response, since the follower layer is the substrate through which the retweets are spread^28, 29^.

In order to establish the relationship between an individual’s features and the response of the system, we have relied on the Independent Cascade (IC) diffusion model. We have formalized the IC model by means of the binomial distribution and a set of assumptions based on empirical evidence (see “Methods”), obtaining the following expression for the response distribution to single actions conditioned on the number of followers (*F*) of the individual:

15 $$\begin{aligned} p(r|\vec {s}) = p(r|F) = B(r;F,p_{inf}) \end{aligned}$$

Where *B*(*x*; *n*, *p*) is a binomial distribution. The discrete joint probability distribution for *A* and *R* is given by:

16 $$\begin{aligned} p(R,A) = p(A) \sum _{F=0}^\infty B(R;AF,p_{inf}) p(F) \end{aligned}$$

The PDF of efficiency is obtained by plugging (16) in (18) and (19).

Notice that *F* is the only component of the feature vector $\vec {s}$ of the individual. The infection probability parameter $p_{inf}$ has been considered constant and equal for every individual and has been determined by Maximum Likelihood Estimation (MLE) of the *p*(*r*) distribution. The discrete computation of the DiA model also requires a fit for the *p*(*F*) distribution, which was performed with the hybrid methodology detailed in the SI. The *p*(*A*) was fit to a discrete truncated power law.

A Monte-Carlo simulation of the DiA model can be performed as follows: Generate a set of individuals with a random number of followers $F \sim p(F)$ and a random activity $A \sim p(A)$. Then, for each action *j* ($j=1,2,\dots ,A$) performed by an individual, the partial response of the system $r_j$ is computed with (15) and the aggregate response with $R=\sum _{j=1}^A r_j$.

For this model, we have found that an analytical derivation of the PDF of efficiency is too cumbersome to be tackled.

**Figure 2:**
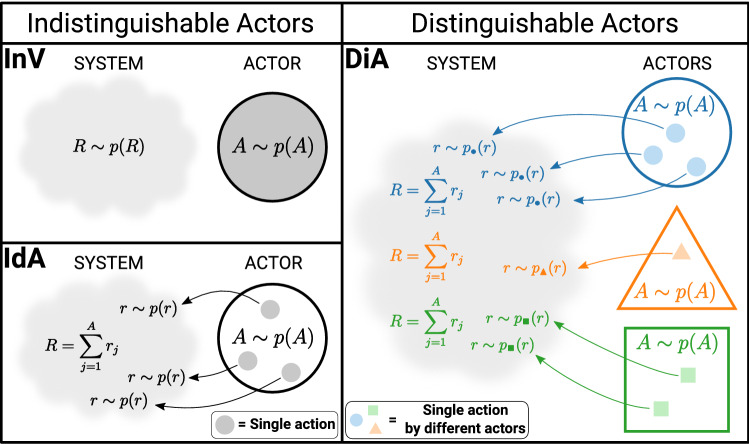
Diagram that summarizes the main characteristics of the three models: Independent Variables (InV), Identical Actors (IdA) and Distinguishable Actors (DiA).

To conclude this section, we summarize the main features of the three developed models in Fig. 2. The models can be classified taking into account two properties: the distinguishability of the actors and the dependence of *R* with respect to *A*. Concerning the distinguishability of the actors, we have on one side the InV and IdA models, where the actors are considered identical, and on the other side the DiA model, where the particular features of the actors are taken into account. Regarding the $A-R$ dependence, we have on one side the InV model, in which *R* and *A* are independent variables, and on the other side, the IdA and DiA models, where *R* depends on *A* because the aggregate response *R* is the sum of the partial responses *r* to each individual action.

### Application of the models to empirical data

The models presented in the previous section have been tested in three different systems: the scientific citations network, Twitter and Wikipedia. See the Supplementary Information (SI) for a detailed description of the datasets. In this section we analyze the models’ performance in each of them.

#### Independent variables model

**Figure 3:**
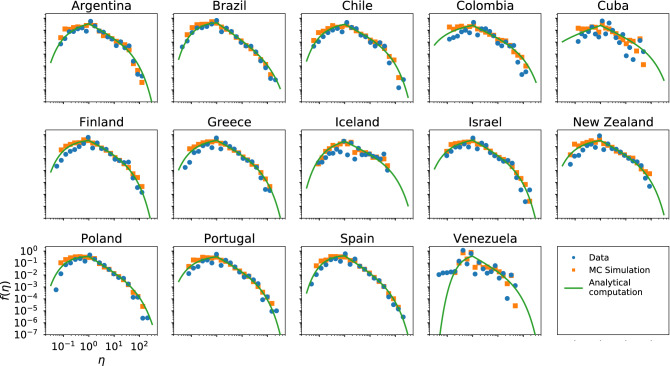
Efficiency distributions corresponding to the InV model applied to the scientific citations dataset. The plots show the empirical efficiency distribution (blue dots), the Monte-Carlo simulation (orange squares) and the analytical expression of (6) (green line).

**Figure 4:**
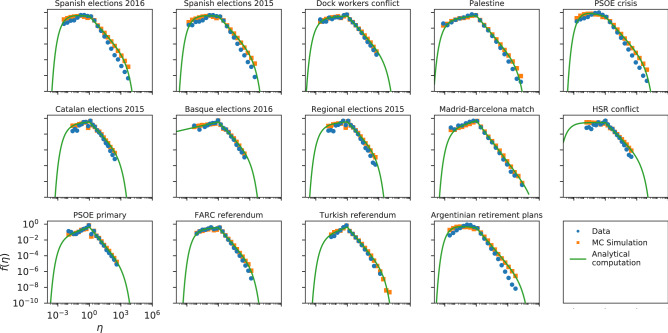
Same as Fig. 3 for the InV model applied to the Twitter datasets.

**Figure 5:**
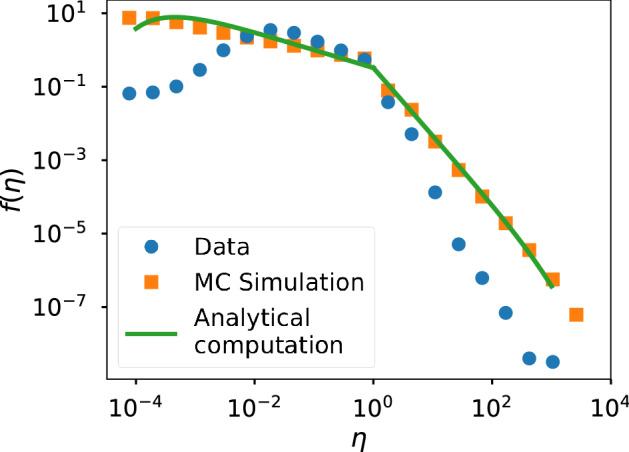
Same as Fig. 3 for the InV model applied to the Wikipedia dataset.

In Figs. 3, 4 and 5 we present the empirical and theoretical (Monte-Carlo simulation and analytical expression) efficiency distributions according to the InV model for scientific citations, Twitter and Wikipedia respectively. In the case of the scientific citations datasets the model adequately reproduces the efficiency distribution in most cases; in particular, we obtain very good fits for the datasets of Brazil and Spain, which are the largest (see SI).

As can be seen in Fig. 4, the InV model captures the general shape of the Twitter efficiency distributions. Nevertheless, on closer examination we see that for several datasets the model does not fully agree with the empirical data, especially in the right tails (see Spanish elections 2015/2016, PSOE crisis or Argentinian retirement plans for example), although for a few others the concordance is quite good (see Madrid–Barcelona match or PSOE primary). Besides, the analytical expression of (6) shows a very good agreement with the Monte-Carlo computation in every case, validating the approximations adopted for the analytical derivation.

The efficiency distribution of the InV model presents a partial agreement with the Wikipedia data in the left tail (see Fig. 5), but a deviation can be appreciated in the lowest values of efficiency and in the right tail. On the other hand, the analytical expression of (6) shows a good concordance with the Monte-Carlo simulation.

Summing up, the InV model captures the universal structure of the efficiency distribution. Furthermore, it reproduces with good accuracy the empirical data of the scientific citations network. However, we achieve slightly worse agreements for several of the Twitter datasets and obtain a higher discrepancy between model and data for Wikipedia. This difference in performance may arise from the long reaction times of citations and prolonged lifespan of scientific publications (months or years)^30^, which contrast with the short characteristic times of the interactions in social media (in the order of minutes or hours)^31–33^. Another important factor to take into account is that while the actions (and the corresponding reactions) in the case of Twitter and Wikipedia are associated to a specific individual because a tweet or an edition have one author (who is the recipient of the retweets and incoming messages), a scientific paper usually has several authors and its associated citations are assigned to all of them. Moreover, as mentioned above, there are many and varied circumstances that determine the citation counts of publications. Therefore, the independence assumption may work for the scientific citations datasets because the different overlapping factors discussed above mask the dependence between *R* and *A*.

Conversely, the model-data discrepancies observed in the other systems may emerge because in that case the independence assumption is not fully adequate. To verify this hypothesis, we check if the $A-R$ correlations neglected by the independence assumption affect the quality of the fit given by the InV model: first, we compute the empirical Spearman’s rank correlation $\rho _e$^34^ between *R* and *A*. Then, we measure the discrepancy $\Delta _{InV}$ between the InV model and the data. Finally, we test if $\rho _e$ and $\Delta _{InV}$ are positively correlated.

The disagreement between the InV model and the data mostly lie on the extreme values of the right tail of the efficiency distribution, which have very low probabilities. Since the right tail can be approximated by a power law, we define $\Delta _{InV}$ as the difference between the theoretical exponent ($\gamma _{InV}=\gamma _R$ from equation (4)) and empirical exponent ($\gamma _{emp}$) of that power law:

17 $$\begin{aligned} \Delta _{InV} = \gamma _{emp} - \gamma _{InV} = \gamma _{emp} - \gamma _{R} \end{aligned}$$

The power law exponent reflects the global trend including the effect of the tail, so the contributions to the error of low probability values are not underestimated. See the SI for more details in this respect.

The relationship between $\Delta _{InV}$ and the empirical correlation $\rho _e$ has been tested by means of a linear regression carried out with the Twitter datasets. This regression (shown in Fig. S2 of the SI) yields a positive correlation of $r=0.80$, indicating that $\Delta _{InV}$ increases monotonously with $\rho _e$ and corroborating the hypothesis presented above.

#### Identical actors model

**Figure 6:**
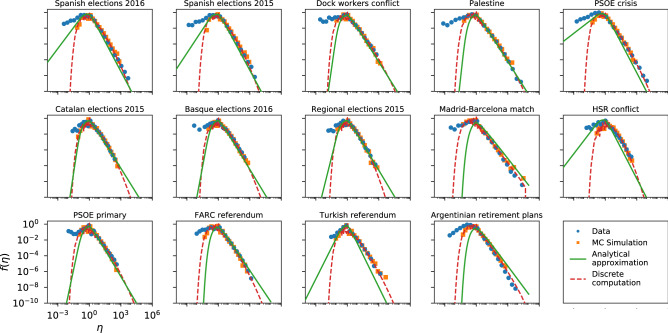
Efficiency distributions corresponding to the IdA model applied to the Twitter datasets. The plots show the empirical efficiency distribution (blue dots), the Monte-Carlo simulation (orange squares), the discrete computation (dashed red line) and the analytical expression of (11) (green line).

**Figure 7:**
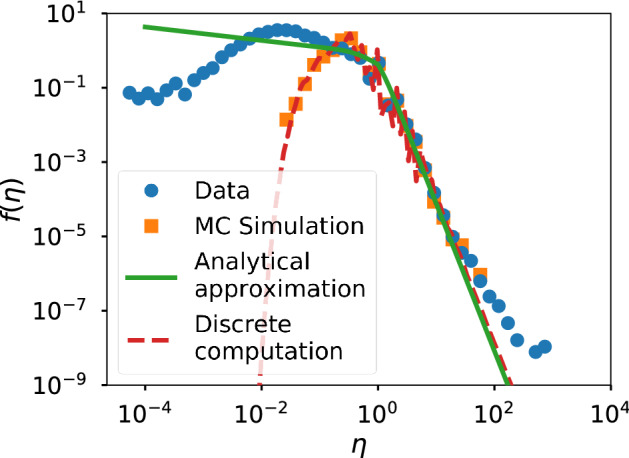
Same as Fig. 6 for the IdA model applied to the Wikipedia dataset.

**Figure 8:**
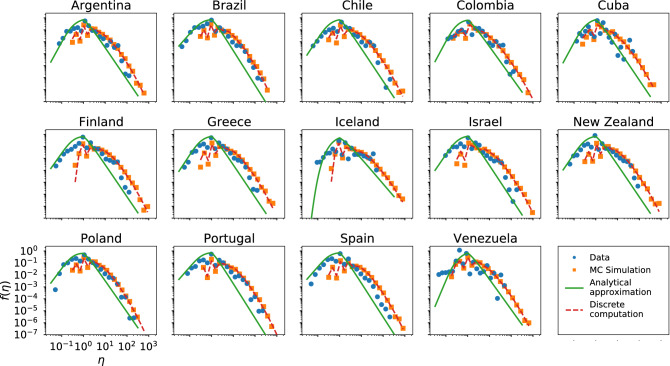
Same as Fig. 6 for the IdA model applied to the scientific citations datasets.

Since the $A-R$ correlations seem to be the cause of the discrepancy between the InV model and the data, we expect that the dependence between *R* and *A* introduced by the IdA model improves the previous results for Twitter and Wikipedia. In Figs. 6, 7 and 8 we present the efficiency distributions for Twitter, Wikipedia and scientific citations according to the IdA model. A clear improvement in the agreement between theory and data can be appreciated on the right tails of all the Twitter datasets shown in Fig. 6. However, there is a higher discrepancy in the left tail of the distribution, which will be discussed below.

If we compare the results of the model got by the analytical expression given by (11) and the discrete computation we find a good correspondence in general. However, there are small deviations that can be explained by the approximations concerning the power-law fit of *p*(*r*) (see SI for details).

In Fig. 7 we can see that for Wikipedia the IdA model is also capable of reproducing the right tail of the distribution adequately. The left tail however falls too fast in this model.

Concerning the scientific citations, the IdA model (in Fig. 8) presents a slightly worse agreement in comparison with the InV model. Therefore, the scientific citations network is better characterized by a model in which activity and response are considered independent. The reasons were discussed in the previous section.

In the IdA model, contrasting with the InV model, *R* and *A* are correlated; but there is no guarantee that the theoretical correlations match the empirical ones. To verify this, we have carried out a linear regression (see Fig. S3 of the SI) between the theoretical Spearman’s correlation $\rho _t$ (averaged from 300 Monte-Carlo realizations of the IdA model) and the empirical one $\rho _e$ for the Twitter datasets, obtaining an $r^2=0.88$ and the following equation for the line: $\rho _t = (1.30\pm 0.14) \rho _e - (0.13\pm 0.06)$. As it can be appreciated, there is a significant correspondence between empirical and theoretical correlations: the slope is close to 1 and the value of the intercept is close to 0, corroborating the ability of the IdA model to reproduce the correlations of the real data to a reasonable extent.

Summarizing, there is an excellent agreement between the IdA model and the data in the right tail of the efficiency distribution for Twitter and Wikipedia. Besides, the correlations induced by the model present a high correspondence with the empirical correlations between *A* and *R*. However, the left tail of the efficiency distribution falls faster in the model than in the data. A low efficiency implies that an individual performs many actions obtaining a very low aggregate response (high *A* and low *R*), but under the IdA model, which considers that *every individual and every action have identical capability* to trigger a response on the system, low efficiencies are very unlikely, as performing many individual actions guarantee at least a moderate aggregate response.

Rather, the empirical evidence suggests that individuals from the left tail of the efficiency distribution ($\eta < 1$) have lower capabilities to trigger a response than those of the right tail ($\eta >1$). This is backed by the fact that the InV model, which considers response independent from activity, reproduces the left tail of the efficiency distribution with better accuracy than the IdA model. Therefore, we hypothesize that the response associated to actors with lower efficiencies do not depend on their activity (so they should show low $A-R$ correlation), contrasting with the users with higher efficiencies, whose behavior can be characterized with the IdA model and should present high $A-R$ correlation.

**Figure 9:**
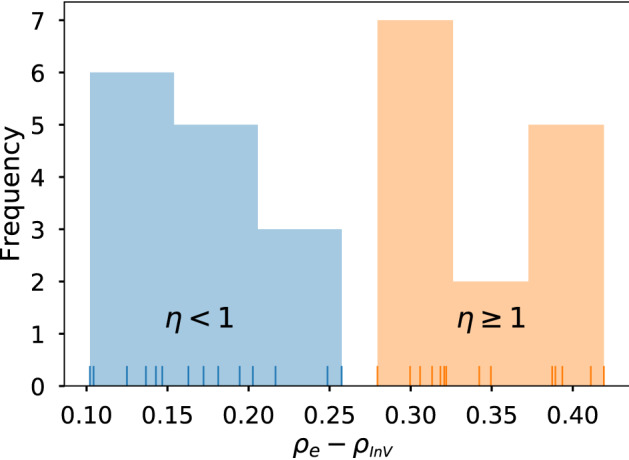
Histogram of the Spearman’s rank correlation between *A* and *R* for the Twitter datasets computed for individuals with low efficiency ($\eta <1$) and high efficiency ($\eta \ge 1$). The small vertical lines in the bottom of the x axis are a rug plot and represent the value of $\rho _e-\rho _{InV}$ for each individual dataset.

The previous hypothesis can be verified by comparing the Spearman’s rank correlation between *A* and *R* for individuals with $\eta <1$ and $\eta >1$. However, we should be careful and take into account the artificial correlations induced by performing this filtering. This is achieved by subtracting the correlation associated to the InV model (averaged from 300 realizations). We have performed this computation for the Twitter datasets and, as can be appreciated in Fig. 9, there is a clear difference between both sets of individuals.

#### Distinguishable actors model

**Figure 10:**
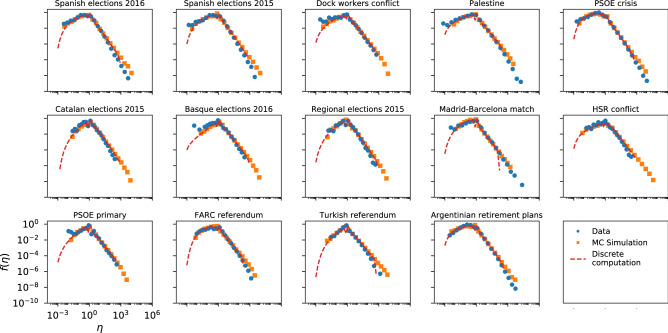
Efficiency distributions corresponding to the DiA model applied to the Twitter datasets. The plots show the empirical efficiency distribution (blue dots), the Monte-Carlo simulation (orange squares) and the discrete computation (dashed red line).

As discussed above, on the one hand, actors with low efficiency have a small impact on the system. Therefore, the InV model, where the interaction between system and individual is *weak* (because the system’s response is not influenced by the individual’s activity) explains their distribution better. On the other hand, actors with high efficiency have a greater impact on the system, so it is the IdA model, where the interaction between individual and system is *strong*, the one that explains their distribution better. Since two models with different assumptions explain different intervals of the efficiency distribution and users with different efficiencies also present different levels of correlation between *A* and *R*, we realize that the response of the system should depend on the features of each actor; that is, we should apply the DiA model. In Fig. 10 we present the results of applying the DiA model to the Twitter datasets by considering the number of followers as the feature that characterizes each actor. It can be appreciated that the DiA model behaves as expected and is able to reproduce both branches of the efficiency distribution for every Twitter dataset.

Additionally, we have carried out a linear regression between the theoretical correlations $\rho _t$ induced by the DiA model and the empirical ones $\rho _e$ (see Fig. S4 of the SI), obtaining a significant correlation between them ($r^2=0.73$). The equation of the resulting line is $\rho _t = (1.03\pm 0.18)\rho _e-(0.13\pm 0.08)$. Notice that the slope is almost 1 and is consistent with that value according to the standard error, indicating that the model reproduces the empirical correlations very faithfully.

The previous results confirm that the DiA model shows a very good agreement between theory and data both for the efficiency distribution and for the $A-R$ correlations. We conclude that in the case of Twitter it is necessary to consider the actors distinguishable to correctly describe the relationship between *A* and *R*.

## Discussion

We have studied the relation between individual actions and the corresponding stimulated collective responses in social systems. To provide a deeper understanding of the systems, we have implemented three models that capture the essence of the dynamics between the two variables. For each model we consider a different level of dependence between both magnitudes. We have used the distribution of the efficiency metric to relate activity *A* and response *R* and evaluate the models in three social systems of different nature: Twitter conversations, the scientific citations network and the Wikipedia collaboration environment. The theoretical efficiency distribution was computed using three methodologies: Monte-Carlo simulation, direct computation with discrete probability distributions and, for two of the models, we have derived analytical expressions.

In a previous work^16^ it was found that the efficiency distribution was independent with respect to the *p*(*A*) distribution, so it was hypothesized that *R* was not affected by *A*. Following this line of reasoning, we developed the Independent Variables model to test if the universal structure of the efficiency distribution could be explained by the efficiency being the ratio of two independent random variables (*R* and *A*) with heterogeneous distributions. We showed that to be true for the general shape but not when finer details are taken into account (some Twitter datasets present discrepancies between data and model in the right tails). Another relevant finding is that our analytical derivation of the InV model also explains the previous empirical findings regarding the independence of the efficiency distribution with respect to the activity distribution^16^. Besides, among the systems under study, the scientific citations data show the highest agreement with the InV model.

By studying the discrepancies in the right tails of the Twitter datasets for the InV model, we found that they could be explained by the presence of correlations that were unaccounted for by the InV model. Following these results, in the Identical Actors model, although all individuals are considered identical, the collective response of the system *R* depends on the number of actions *A* performed by an actor. When this model is tested on empirical data, we obtain excellent fits for the right tail of the efficiency distribution in the Twitter and Wikipedia datasets. Moreover, the IdA model reproduces the empirical correlations between *A* and *R* to a reasonable extent. However, the InV model showed a better agreement than the IdA model on the left tail of the efficiency distribution.

Since two models with different assumptions explained different intervals of the efficiency distribution, we hypothesized that the individual features of the actors must be considered to fully explain their efficiency, leading us to the development of the Distinguishable Actors model, in which the system’s response depends on the characteristics of the individual that performs the action. We have applied this model to Twitter data by considering the number of followers of a user as a proxy of her ability to trigger a response in the system. Finally, we have shown that the DiA model presents a very good agreement with both tails of the empirical distribution of efficiency for the Twitter datasets and faithfully reproduces the empirical correlations between *R* and *A*.

To conclude, we would like to stress that the adopted modeling approach, based on Ockham’s razor principle, endows the models with a high explanatory power and provides fast and simple ways to compute them. It is also worth emphasizing the usefulness of the analytical expressions developed for the InV and IdA models, which enable immediate calculations. Although the developed formalism is general and domain-independent, the aforementioned properties also make them suitable to be used as null models for more elaborated and domain-specific approaches.

## Methods

### Estimation of the PDF of efficiency from the discrete joint probability distribution of *A* and *R*

In order to obtain the efficiency distribution from the discrete computations of the IdA and DiA models we start from the cumulative probability distribution of efficiency:

18 $$\begin{aligned} P(H \le \eta ) = \sum _{A=1}^\infty \sum _{R=0}^{\lfloor A \eta \rfloor } p(R,A) \end{aligned}$$

where $\lfloor \cdot \rfloor$ is the floor operator. Then, average probability densities for efficiency intervals $[\eta _a, \eta _b]$ can be computed as:

19 $$\begin{aligned} \bar{f}(\eta \in [\eta _a, \eta _b]) \approx \frac{P(H \le \eta _b)-P(H \le \eta _a)}{\eta _b - \eta _a} \end{aligned}$$

We adopt this approach to be able to compare our theoretical distributions with the empirical histograms.

### Analytical approximation of the IdA model

An analytical solution of the IdA model can be obtained if one is able to find an expression for the sum of *A* independent random variables $\{r_1,r_2,\dots ,r_A\}$ following the same distribution *p*(*r*); that is, an expression for the $A-fold$ convolution of *p*(*r*) with itself.

Although finding an analytical expression for the sum of random variables is not always feasible, there exist approximations that work in some cases. We have modeled *p*(*r*) as a power law and adopted an approximation proposed by Zaliapin et al.^35^ to obtain the distribution of the sum of power-law distributed random variables. This approximation consists in replacing the sum by the maximum summand $R = \sum _{i=1}^A r_i \approx \max (\{r_i\}_{i=1}^A)$. In that case, if the PDF of *r* is:

20 $$\begin{aligned} f_r(r) = (\gamma _r -1)r_m^{\gamma _r-1} r^{-\gamma _r} \end{aligned}$$

The conditional cumulative distribution function of *R* given *A* can be computed as:

21 $$\begin{aligned} F(R|A) \propto e^{-( R^{1-\gamma _r} A)} \end{aligned}$$

Then, if the distribution of *A* is modeled as a power law with the form $p(A)\propto A^{\gamma _A}$ and we assume that the minimum values of activity and response are $A_m =1$ and $R_m = 1$ (which is what is observed for every dataset), the approximated joint probability density is:

22 $$\begin{aligned} \varphi (R,A) = \frac{(\gamma _r-1)(\gamma _A-1)}{1+(1-\gamma _A)\Gamma (1-\gamma _A,1)} A^{1-\gamma _A}R^{-\gamma _r}e^{-AR^{1-\gamma _r}} \end{aligned}$$

Plugging this in (2), we obtain the expression of (11).

In principle, the expression of (21) proposed in^35^ is valid when $\gamma _r < 2$. However, we have found that the expression of (11) also provides a good fit for the data when $\gamma _r>2$, although the agreement quickly deteriorates when $\gamma _r$ exceeds a value of around 2.5.

### Formulation of the independent cascade model to compute the DiA model

In the case of Twitter, the ability of the individuals to trigger a response depends mainly on their location on the follower network^29^, meaning that it is a topocratic network^36^. In a previous work^16^ it was also shown that the Independent Cascade (IC) model can be used to reproduce the efficiency distribution. Hence, in order to determine the $p(r|\vec {s})$ distribution, we will rely on that application of the IC model to the Twitter follower network.

Taking into account that most information cascades in Twitter are shallow^28, 37^, we have performed a first-neighbors approximation and focused on the response generated only in the first layer of diffusion. In that scenario, when a node becomes active (publishes a tweet), each follower can be *infected* with a probability $p_{inf}$, which we consider constant. If the active node has *F* followers, this process can be formalized as *F* Bernoulli trials (coin tosses) with success probability $p_{inf}$. Then, the response *r* to this single action is a random variable with binomial distribution:

23 $$\begin{aligned} p(r|F) = B(r;F,p_{inf}) \end{aligned}$$

The computation of the distribution of aggregate response *R* is straight forward, as the sum of two binomial variables ($y=x_1+x_2$) with $n_1$ and $n_2$ trials and same success probability *p* also follows a binomial distribution of the form $B(y;n_1+n_2,p)$. Therefore, we can consider that, if for a single action there are *F* coin tosses, for *A* actions we have *FA* trials, obtaining the following distribution:

24 $$\begin{aligned} p(R|F,A) = B(R;FA,p_{inf}) \end{aligned}$$

Notice that $p_{inf}$ can be considered as an effective infection probability that includes the effect of the higher layers. We have determined $p_{inf}$ by fitting the empirical single-action response distribution *p*(*r*) to the corresponding theoretical distribution for this model through MLE:

25 $$\begin{aligned} p(r) = \sum _{F=0}^\infty B(r;F,p_{inf})p(F) \end{aligned}$$

where *p*(*F*) is the follower distribution. Once the $p_{inf}$ has been determined the joint probability distribution *p*(*R*, *A*) can be computed as:

26 $$\begin{aligned} p(R,A) = p(A) \sum _{F=0}^\infty B(R;AF,p_{inf}) p(F) \end{aligned}$$

And finally, the efficiency distribution can be determined with (18) and (19). The numerical computations for the DiA model have been carried out with Twitter data considering the following cut-off values for the followers, the activity and the response: $F_{max} = 500{,}000; A_{max} = 1{,}000; R_{max} = 1{,}000$.

Although we have adopted this first-neighbors approximation mainly for simplicity and computational feasibility reasons, in the SI we present a formulation of the IC model that takes into account all the diffusion layers.

## Supplementary information

Supplementary Information.
