# Supplementary material for: Impact of individual actions on the collective response of social systems
Source: Sci Rep. 2020 Jul 22;10:12126. doi: 10.1038/s41598-020-69005-y (PMC7376036; doi:10.1038/s41598-020-69005-y)
Supplement: Supplementary file 1 — Supplementary Information. [file 41598_2020_69005_MOESM1_ESM.pdf]

Supplementary Information for:
Impact of individual actions on the collective response of social systems

S. Martin-Gutierrez, J. C. Losada and R. M. Benito

Contents

1 Description of the datasets **4**
1.1 Twitter datasets 4
1.2 Scientific citations datasets 6
1.3 Wikipedia dataset 7

2 Computing the efficiency distribution with random variable algebra **7**

3 Full Independent Cascade model formalism **8**

4 Hybrid fitting methodology for the $p(r)$ and $p(F)$ distributions **11**

5 Measurement of the discrepancy between the InV model and the data and its relationship with empirical $A - R$ correlations **11**

6 Theoretical and empirical $A - R$ correlations **12**

7 Intermediate computations and fits **12**
7.1 Independent variables model 12
7.1.1 Scientific citations 13
7.1.2 Twitter 13
7.1.3 Wikipedia 13
7.2 Identical actors model 13
7.2.1 Scientific citations 13
7.2.2 Twitter 14
7.2.3 Wikipedia 14
7.2.4 The error associated to the PL fit of $p(r)$ affects the quality of the IdA model's analytical approximation 14
7.3 Distinguishable actors model 15

List of Figures

S1 Diagram showing the regions of integration defined to each side of the line $\eta_o = \frac{r_m}{a_m}$ to obtain the cumulative distribution function of the efficiency. 21

S2 The deviation of the InV model (Δ_{InV}) with respect to the Twitter empirical data is related to the correlations between A and R, measured with the Spearman's correlation (ρ_e). The figure shows a monotonous increment of Δ_{InV} with ρ_e, which has been characterized by means of a linear regression. . . . 22
S3 Linear regression of the correlation induced by the IdA model (blue dots) with respect to the empirical correlation computed for the Twitter datasets. Each point corresponds to a different conversation. For comparison the values corresponding to the InV model have also been plotted (orange squares). . . . 23
S4 Linear regression of the correlation induced by the DiA model with respect to the empirical correlation computed for the Twitter datasets. Each point corresponds to a different conversation. The reference values obtained for the InV model are also displayed. . . . 24
S5 Fit of the empirical distribution of activity of the scientific citations network to a truncated power law. Blue dots correspond to the full data and orange squares to the data selected to perform the fit ($A \in [1, 30]$). . . . 25
S6 Fit of the empirical distribution of response of the scientific citations network to a truncated power law. Blue dots correspond to the full data and orange squares to the data selected to perform the fit ($R \in [2, 300]$). . . . 26
S7 Fit of the empirical distribution of activity of the Twitter datasets to a power law (dashed green line) and to a truncated power law (continuous orange line). . . . 27
S8 Fit of the empirical distribution of response of the Twitter datasets to a power law (dashed green line) and to a truncated power law (continuous orange line). . . . 28
S9 Fit of the right tail of the distribution of efficiency of the Twitter datasets to a power law. . . 29
S10 Fit of the empirical distribution of activity of the Wikipedia dataset to a power law (dashed green line) and to a truncated power law (continuous orange line). . . . 30
S11 Fit of the empirical distribution of response of the Wikipedia dataset to a TPL. . . . 31
S12 Fit of the empirical distribution of activity of the scientific citations network datasets to a PL. 32
S13 Hybrid lognormal fit of the empirical distribution of response to single actions of the scientific citations network datasets. . . . 33
S14 Power law fit of the empirical distribution of response to single actions of the scientific citations network datasets. . . . 34
S15 Hybrid lognormal fit of the empirical distribution of response to single actions of the Twitter datasets. . . . 35
S16 Power law fit of the empirical distribution of response to single actions of the Twitter datasets. 36
S17 Hybrid PL fit of the empirical distribution of response to single actions of the Wikipedia dataset. . . . 37
S18 Power law fit of the empirical distribution of response to single actions of the Wikipedia dataset. . . . 38
S19 Relationship between the fitting error committed when $p(r)$ is modeled as a power law (ϵ_r) and the deviation of the analytical approximation with respect to the numerical computation of the IdA model (ϵ_η) in the right tail of the efficiency distribution $f(\eta)$. . . . 39
S20 Fit of the empirical distribution of followers of the Twitter datasets to the empirical values with a PL tail. . . . 40

List of Tables

S1 Description of the Twitter datasets. . . . 5
S2 Description of the datasets of scientific citations. . . . 7

S3 Parameters of each fit performed with the scientific citations datasets. 17
S4 Parameters of each fit performed with the Twitter datasets. 17
S5 Parameters of each fit performed with the Wikipedia data. 17
S6 Values of p_{inf} estimated through MLE for the Twitter datasets. The averages and standard deviations obtained for 10 samples of 1000 instances each are presented. 18

In the first section of the Supplementary Information we present a detailed description of the datasets. In the second section, we derive the general formalism to compute the efficiency distribution from the activity and response distributions. In the third, the Independent Cascade model is fully formalized considering all the diffusion layers. In the fourth section, we explain the hybrid methodology adopted to fit several empirical probability distributions. In the fifth section we detail the computations carried out to measure the discrepancy between the InV model and the data. In the sixth section we discuss the empirical and theoretical correlations between R and A. Finally, in the seventh section we present the fits of the data that have to be performed in order to compute the models.

1 Description of the datasets

We have worked with 29 datasets of three social systems of different nature: Twitter, the Wikipedia collaboration environment and the scientific citations network. Each dataset is thoroughly described below.

1.1 Twitter datasets

We have worked with 14 datasets extracted from different Twitter conversations. Each dataset has been retrieved by searching for tweets containing one of a given set of keywords during a specific period of time. In table S1 we present the keywords used to build each dataset as well as the number of users that took part in each conversation, the number of tweets they published and the time period considered in each case. From this information we have extracted the number of original tweets published by each user, which corresponds to the activity A, and the number of retweets obtained by each user, which is the response R. We have also computed the number of times each original tweet has been retweeted, which is the response of the system to single actions r.

Dataset	Keywords	No. Tweets	No. Users	Time interval	Time span/days
Spanish elecions 2015	20D, 20D2015, #EleccionesGenerales2015	2779072	200159	2015/11/11 – 2016/05/09	180
Spanish elections 2016	26J, 26J2016, #EleccionesGenerales2016, #Elecciones26J	2814781	174110	2016/05/09 – 2016/11/13	188
Dock workers conflict	estiba, estibadores	218328	13489	2017/05/26 – 2017/07/14	49
Palestine	Gaza, Ġazza, palestine, jerusalem, Yerushaláyim, Yerushalayim, yerussalem, quds, [same words in hebrew and arabic]	3782389	372031	2017/12/20 – 2018/01/11	22
PSOE crisis	psoe	4766675	191260	2016/09/30 – 2016/11/04	35
Catalan elections 2015	27s, 27s2015	90009	8130	2015/09/10 – 2015/09/15	5
Basque elections 2016	25s	97835	8535	2016/09/16 – 2016/09/27	11
Spanish regional elections 2015	24m	202865	20710	2015/05/21 – 2015/05/26	5
Madrid-Barcelona match	madrid barsa, madrid barça, futbol madrid, fútbol madrid, futbol barcelona, fútbol barcelona, futbol barsa, fútbol barsa, fútbol barça, futbol barça, elclasico, elclásico, fcbarcelona, fcbarcelona_es, fcbarcelona_cat, realmadrid, clasico futbol, clásico futbol, clasico fútbol, clásico fútbol, clasico madrid, clásico madrid, clasico barsa, clasico barça, clásico barsa, clásico barça	1394346	205991	2017/12/22 – 2017/12/25	3
Murcia's HSR conflict	soterramientoya, soterramientomu, noalmuromurcia, alasvias, alasvías	129617	4208	2017/10/06 – 2017/11/17	42
PSOE primary	primarias psoe	95874	13914	2017/05/20 – 2017/05/25	5
FARC referendum	colombia acuerdo, colombia tratado,colombia farc, colombia referendum, referendum farc, tratado farc, acuerdo farc, colombia voto, colombia votar	325050	40454	2016/10/07 – 2016/11/03	27
Turkish referendum	Turkeyreferendum	119250	13021	2017/04/15 – 2017/04/18	3
Argentinian retirement plans	reforma argentina, disturbios argentina, pension argentina, pensión argentina, pensiones argentina, protesta argentina, protestar argentina, protestas argentina, violencia argentina, macri, mauriciomacri, cacerola argentina, cacerolas argentina	4194063	265418	2017/12/21 – 2018/02/14	55

Table S1: Description of the Twitter datasets.

Here we provide some information on the contexts of the Twitter datasets:

- Spanish elections 2015: On 20 December 2015 the Spanish general elections were held [1, 2].
- Spanish elections 2016: On 26 June 2016, another general elections were held in Spain due to failure in the government formation process after the previous election [3].
- Dock workers conflict: A labor dispute around the liberalization of the stevedoring activity in Spanish docks [4].
- Palestine: This dataset tries to capture the online conversations regarding the conflict around Palestine.
- PSOE crisis: The Spanish Socialist's Workers Party (Partido Socialista Obrero Español) underwent a crisis that forced its leader dismissal on 28 September 2016 [5].
- Catalan elections 2015: Elections for the regional government of Catalonia held on 27 September 2015 [6].
- Basque elections 2016: Elections for the regional government of the Basque Country held on 25 September 2016 [7].
- Spanish regional elections 2015: Elections for the regional parliaments of thirteen out of the seventeen autonomous communities in Spain and local elections for all the municipalities held on 24 May 2015 [8].
- Madrid-Barcelona match: A football match held the 23 of December 2017 [9] between the two top-teams [10] of the Spanish football league: Real Madrid Club de Fútbol and Barcelona Fútbol Club.
- HSR conflict: Protests in the city of Murcia, Spain, around the building of a wall for the high speed rail (HSR) that would divide the city [11].
- PSOE primary: The election held by the Spanish Socialist's Workers Party to choose new leadership after the crisis of 2016 [12].
- FARC referendum: A referendum to ratify the final agreement on the termination of the Colombian conflict between the Colombian government and the FARC guerillas was held on 2 October 2016. It failed with 50.2% voting against it and 49.8% voting in favor [13].
- Turkish referendum: A constitutional referendum was held throughout Turkey on 16 April 2017 on whether to approve 18 proposed amendments to the Turkish constitution that would replace the existing parliamentary system of government by an executive presidency [14].
- Argentinian retirement plans: A reform of the public retirement plans system in Argentina passed by the government on 19 December 2017 triggered violent protests [15].

1.2 Scientific citations datasets

We have queried the Web of Science [16] to extract all the scientific papers published in several countries as well as their authors and the citation data for both papers and authors. The data spans from 2008 to 2017. Although the authors are associated to a given country, the citations they receive come from any author in any country. In table S2 we show the number of papers and authors for each country. In these datasets, the number of papers published by a given author is her activity A and the total number of citations she has obtained, the response R. The single-action response r in this case corresponds to the number of citations to a given paper.

Dataset	No. Publications	No. Authors
Argentina	89070	56095
Brazil	406493	211829
Chile	68375	53318
Colombia	41106	45983
Cuba	10570	8723
Finland	118223	82957
Greece	106241	71089
Iceland	9623	16725
Israel	133219	75765
New Zealand	86875	54900
Poland	233673	122556
Portugal	122848	85512
Spain	524770	261740
Venezuela	12488	8921

Table S2: Description of the datasets of scientific citations.

1.3 Wikipedia dataset

The Wikipedia dataset has been retrieved from the Stanford Large Network Dataset Collection [17]. It contains data about the editions made by users on the Wikipedia pages from the beginning of the Wikipedia project on 15 January 2001 to January 2008. In this case, we have only one dataset, which corresponds to the English Wikipedia, that includes 250 million registered editions by 2.3 million users. In Wikipedia users can edit regular article pages, the talk pages associated to each article to coordinate the redaction and also personal user pages, that are intended as a way of exchanging direct messages between users. In this system, we have considered the number of editions performed by a user as her activity A and the number of editions made by other users in her personal page as the response R. In this case, there is no direct way to define the response of the system to single actions as in the other two studied systems. Hence, we have defined r as the number of editions made on the personal pages of users that have performed only one edition themselves; that is, users whose activity A is 1.

2 Computing the efficiency distribution with random variable algebra

For the sake of clarity, only for this section we will adopt the usual convention in statistics of writing the name of a random variable with an uppercase letter (X) and a specific value of that variable as the corresponding lowercase letter (x). Therefore, when we write r in this section, we are referring to a specific value of the response random variable R, not to the partial response to a single action by a given actor.

The distribution of efficiency can be obtained from the joint probability distribution of A and R with random variable algebra [18, 19]. Let A and R be two random variables with support $a \in [a_m, \infty)$ and $r \in [r_m, \infty)$ for some $a_m, r_m > 0$. Efficiency (H - uppercase η) is another random variable defined as $H = \frac{R}{A}$. Let η be a particular value of H. The cumulative probability that $H \leq \eta$ can be written as:

$$p(H \leq \eta) = p(\frac{R}{A} \leq \eta) = p(R \leq \eta A) = p(A \geq \frac{R}{\eta}) \tag{S1}$$

Where we have replaced H by R/A. Let us now make the approximation that A and R are continuous variables with joint probability distribution $\varphi_{R,A}(r, a)$ (which we will call $\varphi(r, a)$ to simplify notation). We

can compute the cumulative distribution function (CDF) of H; that is, $F_H(\eta) = p(H \leq \eta)$, by integrating $\varphi(r,a)$ in the region where Eq. (S1) is fulfilled:

$$F_H(\eta) = \begin{cases} \int_{r_m}^{\infty} \int_{r/\eta}^{\infty} \varphi(r,a)dadr & \text{if} \quad \eta \leq \frac{r_m}{a_m} \\ \int_{a_m}^{\infty} \int_{r_m}^{\eta a} \varphi(r,a)drda & \text{if} \quad \eta > \frac{r_m}{a_m} \end{cases} \tag{S2}$$

In figure S1 we show a diagram explaining the regions of integration. Notice that in the two-dimensional (r,a) space, the subspaces of constant efficiency are straight lines that pass through $(0,0)$ because, for a specific efficiency $\eta_* \neq 0$, we can write $a = \frac{1}{\eta_*}r$. Therefore, lines with lower η_* have higher slope and vice versa. The probability that the efficiency takes a value smaller than a given η_*; that is, $p(H \leq \eta_*)$, can be found by integrating the probability density $\varphi(r,a)$ to the *left* of the $a = \frac{1}{\eta_*}r$ line (integrate for all the efficiencies $\eta \leq \eta_*$, so lines with higher slope than $1/\eta_*$). Now, because the minimum values a_m, r_m are larger than 0, the regions of integration must be defined differently to each side of the line with $\eta_o = \frac{r_m}{a_m}$ (thick dashed line of the diagram). The green area is an example of area of integration associated to a particular efficiency value $\eta_1 < \eta_o$. There, the integral in variable a runs from the line defined by $a = \frac{1}{\eta_1}r$ to infinity and in variable r from r_m to infinity. When we integrate for a efficiency value $\eta_2 > \eta_o$, the area of integration, an example of which is shown in orange, is defined by taking variable r running from r_m to the line $r = \eta_2 a$ and variable a from a_m to infinity. If we used integration regions of the *green kind* in the area where $\eta > \eta_o$, we would incorrectly include the red area in the integral, while if we used integration regions of the *orange kind* in the area where $\eta < \eta_o$, we would incorrectly include the yellow area in the integral. Therefore, we must compute $F_H(\eta)$ differently to each side of $\eta_o = \frac{r_m}{a_m}$ to obtain the right result. The two branches of Eq. (S2) correspond to the two tails of the efficiency distribution.

Once we have the cumulative distribution function $F_H(\eta)$, the Leibniz integral rule can be used to get $f_H(\eta) = \frac{dF_H(\eta)}{d\eta}$. For $\eta \leq \frac{r_m}{a_m}$:

$$\begin{gathered} f_H(\eta) = \frac{d}{d\eta}\left[\int_{r_m}^{\infty} \int_{r/\eta}^{\infty} \varphi(r,a)dadr\right] = \int_{r_m}^{\infty} \varphi(r, \frac{r}{\eta})\left(\frac{r}{\eta^2}\right)dr \\ \text{Change of variables} \quad r = \eta a \quad \Rightarrow \quad f_H(\eta) = \int_{r_m/\eta}^{\infty} \varphi(\eta a, a)ada \end{gathered} \tag{S3}$$

And for $\eta > \frac{r_m}{a_m}$:

$$f_H(\eta) = \frac{d}{d\eta}\left[\int_{a_m}^{\infty} \int_{r_m}^{\eta a} \varphi(r,a)drda\right] = \int_{a_m}^{\infty} \varphi(\eta a, a)ada \tag{S4}$$

We use Eq. (S3) and Eq. (S4) to get the analytical expressions of the InV and IdA models. They are rewritten in Eq. (2) of the main document dropping the uppercase/lowercase convention adopted only for this section, so R is used instead of r and A is used instead of a.

3 Full Independent Cascade model formalism

In this section, we develop the formalization of the Independent Cascade model adopted to compute the DiA model for the Twitter data. Here we start by considering all the diffusion layers and then carry out the first-neighbors approximation used for the computations presented in the main document.

The IC model consists in a diffusion mechanism [20] that can be outlined as follows. Assume that we have a set of agents (nodes) that are connected with a set of links forming a static directed network (the links do not change through time). Choose one node randomly to be activated. The active node tries to activate each of her neighbors one by one. There is only one attempt per neighbor and all the attempts are independent. The success probability for each activation attempt depends on the link that joins the source

node and the destiny node. The successfully *infected* nodes become active and try to activate their neighbors. This process is iterated until the diffusion cascade dies out.

Now, drawing on the work by Morales et al. [21], let us assume that the probability of a given node to activate her neighbors only depends on her distance to the source of the cascade. If she is the source, the *infection* probability will be p^1_{inf}. If she is l layers away from the source, the infection probability will be p^l_{inf}. The number of *neighbors* of a node is her out-degree k^l_{out} (considering the direction of the links as the direction of propagation of influence), which we will call k^l in order to simplify the notation.

Taking the above into account, let us try to find the response distribution conditioned on the activity. Notice that if we look at a given active node trying to *infect* her neighbors we can model this process with a binomial variable: In the first layer, the node has k^1 Bernoulli trials to activate a neighbor and each trial has a success probability p^1_{inf}. The number of successfully activated neighbors in that layer corresponds to the response r^1, which can take values between 0 and k^1. The response distribution for the first layer of the diffusion cascade would be

$$p(r^1|k^1) = B(r^1; k^1, p^1_{inf}) \tag{S5}$$

With $B(x; n, p)$ a distribution of a binomial variable x with n Bernoulli trials and p success probability.

An individual that performs A actions and has k^1 neighbors will trigger an aggregated response $R^1 = \sum_{i=1}^{A} r^1_i$ in her immediate neighbors (the first layer). The distribution of the aggregated response can be computed straight forward, as the sum of two binomial variables ($y = x_1 + x_2$) with n_1 and n_2 trials and same success probability p also follows a binomial distribution of the form $B(y; n_1 + n_2, p)$. The probability distribution for R^1 given a specific activity A, an out-degree k^1 and an infection probability p^1_{inf} is then:

$$p(R^1|A, k^1) = B(R^1; Ak^1, p^1_{inf}) \tag{S6}$$

Our next approximation is that a response of R^1 implies that R^1 nodes were activated (the reality is that there were R^1 activations of a total of k^1 nodes, some of them possibly activated more than once). The R^1 active nodes have $k^2_1, k^2_2, \ldots, k^2_{R^1}$ neighbors (the superscript 2 indicates that they are in the second diffusion layer). Each of the active nodes tries to *infect* her neighbors with the same infection probability p^2_{inf}. Hence, there are a total of $k^2_T(R^1) = \sum_{i=1}^{R^1} k^2_i$ Bernoulli trials with success probability p^2_{inf}, resulting in the following distribution of aggregated response for the second diffusion layer:

$$p(R^2|R^1, k^2_T(R^1)) = B(R^2; k^2_T(R^1), p^2_{inf}) \tag{S7}$$

We can marginalize this with respect to R^1 and $k^2_T(R^1)$ in order to get the distribution of R^2, but first we need to obtain the distribution of $k^2_T(R^1)$, which can be computed as the $R^1 - fold$ convolution of the empirical $p(k)$ distribution with itself:

$$p(k^2_T|R^1) = p^{*R^1}(k) \tag{S8}$$

In order to be able to do this, we have to assume that $p(k^{l+1}|k^l) = p(k)$; that is, that there exists no degree correlations. This is a reasonable approximation for Twitter because it has been observed that the degree correlations are small ($|\rho| < 0.2$) [22, 2, 23], a property that is also present in other online social networks [24].

The probability distribution of R^2 is finally:

$$
\begin{aligned}
p(R^2|A,k^1) &= \sum_{k_T^2=0}^{\infty}\sum_{R^1=0}^{\infty} p(R^2|R^1,k_T^2(R^1))p(k_T^2|R^1)p(R^1|A,k^1) \\
&= \sum_{k_T^2=0}^{\infty}\sum_{R^1=0}^{\infty} B(R^2;k_T^2(R^1),p_{inf}^2)p^{*R^1}(k)B(R^1;Ak^1,p_{inf}^1)
\end{aligned}
\tag{S9}
$$

This process can be iterated to get the response distribution for layer l as a function of the one obtained in layer $l-1$:

$$
\begin{aligned}
&p(R^l|A,k^1) = \\
&= \sum_{k_T^l=0}^{\infty}\sum_{R^{l-1}=0}^{\infty} p(R^l|R^{l-1},k_T^l(R^{l-1}))p(k_T^l|R^{l-1})p(R^{l-1}|A,k^1) \\
&= \sum_{k_T^l=0}^{\infty}\sum_{R^{l-1}=0}^{\infty} B(R^l;k_T^l(R^{l-1}))p^{*R^{l-1}}(k)p(R^{l-1}|A,k^1)
\end{aligned}
\tag{S10}
$$

The total aggregated response triggered in all the layers would be $R = \sum_{l=1}^{\infty} R^l$. Hence, the distribution of the response given an activity A and a number of neighbors k^1 is the convolution of the distributions of response for each layer:

$$
p(R|A,k^1) = p(R^1|A,k^1) * p(R^2|A,k^1) * \cdots \tag{S11}
$$

Finally, the joint probability distribution for R and A can be obtained as:

$$
p(R,A) = p(A)\sum_{k^1=0}^{\infty} p(R|A,k^1)p(k^1) \tag{S12}
$$

Notice that $p(k^1) = p(k)$. In this model, the infection probabilities $\{p_{inf}^l\}_{l=1}^{\infty}$ are free parameters that must be determined in some way, be it by direct computation if the data is available, by Maximum Likelihood Estimation or any other fitting technique.

The computations in this model comprehend an infinite number of diffusion layers, making it numerically intractable. We have chosen, as a first order approximation, to consider only the response of the first layer. The rationale behind this approximation is that it has been observed that most information cascades in Twitter are shallow [25, 26]. Moreover, the computations involved for the second and farther layers are very demanding in terms of memory and computation time, and the results that we obtain for the first-layer approximation are already in good agreement with the data.

Since we work with the follower network of Twitter, the out-degree distribution $p(k)$ corresponds to the follower distribution $p(F)$. Hence, for our first-layer approximation to the independent cascade model, the distribution of response R given the activity A and a certain number of followers F is:

$$
p(R|A,F) = B(R;AF,p_{inf}) \tag{S13}
$$

Where we use an effective infection probability p_{inf} that includes the effect of the higher layers. We have determined p_{inf} by fitting the empirical distribution of response to single actions $p(r)$ to the corresponding theoretical distribution for this model with MLE:

$$
p(r) = \sum_{F=0}^{\infty} B(r;F,p_{inf})p(F) \tag{S14}
$$

Where $p(F)$ is the empirical follower distribution. Once the p_{inf} has been determined it is straight forward to compute the joint probability distribution $p(R, A)$:

$$p(R, A) = p(A) \sum_{F=0}^{\infty} B(R; AF, p_{inf}) p(F) \tag{S15}$$

4 Hybrid fitting methodology for the $p(r)$ and $p(F)$ distributions

The hybrid methodology has been developed to fit several distributions in order to obtain the most faithful reproduction of the data, such that the errors committed when performing numerical computations are minimized.

Since the empirical distributions of discrete variables k analyzed in this work present a heavy tail (see SI), the highest probabilities are associated to low values of k, where the empirical distribution is very well defined. The smaller probabilities, which correspond to high values of k, show a more noisy behavior due to a lack of data. Also, beyond a certain threshold there are no more data points.

Taking this into account, the empirical distribution has been fit by taking the raw empirical values p_k^{emp} of the probabilities for low k and then fitting the tail of the distribution to a theoretical one with probabilities p_k^{fit}. In particular, the lognormal and the truncated power law distributions have been employed. The tail is defined as those values of k which are larger than a given threshold q. The resulting hybrid distribution has the following form:

$$p(k) = \begin{cases} p_k^{emp} & if \quad k \leq q \\ C p_k^{fit} & if \quad k > q \end{cases} \tag{S16}$$

Where the normalization constant C can be obtained by properly normalizing $p(k)$:

$$C = \sum_{k=q}^{\infty} p_k^{emp} \tag{S17}$$

With this methodology a very faithful representation of $p(k)$ is obtained and we are able to compute the probabilities for values of k that are missing in the data; in particular, very high and unlikely values.

The distribution of response to single actions has been modeled with this methodology using a lognormal and the follower distribution has been fit employing a truncated power law for the tail. The resulting fits are presented below.

5 Measurement of the discrepancy between the InV model and the data and its relationship with empirical $A - R$ correlations

The discrepancy between the InV model and the data can be characterized by the disagreement in the right tails of the efficiency distributions. This disagreement has been determined by modeling the right tail of the empirical efficiency distribution as a power law ($p(\eta) \propto \eta^{-\gamma_{emp}}$) and computing the difference between the empirical γ_{emp} exponent and the γ_{InV} exponent according to the InV model. For this purpose, the response distribution was modeled as a power law with exponent γ_R. As we discussed in the main document, in that case $\gamma_{InV} = \gamma_R$. Consequently, the measure for the deviation between the InV model and the empirical results can be defined as:

$$\Delta_{InV} = \gamma_{emp} - \gamma_{InV} = \gamma_{emp} - \gamma_R \tag{S18}$$

In figure S2 we plot the error associated to the InV model Δ_{InV} against the empirical Spearman's correlation between A and R (ρ_e) and each point corresponds to a different Twitter conversation. It can be appreciated that Δ_{InV} grows monotonously with ρ_e.

6 Theoretical and empirical $A - R$ correlations

To show that the IdA and DiA models reproduce the empirical $A - R$ correlations we generate 300 MC simulations of the models for each Twitter dataset and for every simulation we compute the corresponding $A - R$ correlation (ρ_t). Then we compute the mean and the standard deviation of the 300 MC simulations to get a confidence interval for the theoretical correlations.

In figure S3 we plot the theoretical Spearman's correlation according to the IdA model (ρ_t) against the empirical one (ρ_e) for the Twitter datasets and carried out a linear regression between both magnitudes. As it can be appreciated, there is a significant correlation between the empirical and the theoretical correlations. Moreover, the slope is close to 1 and the value of the intercept is close to 0. This confirms that the IdA model reproduces the correlations of the real data to a reasonable extent.

The correlations that the DiA model induces between A and R (ρ_t) have been represented against the empirical correlations (ρ_e) in figure S4. The linear regression that is included in the aforementioned figure shows that, analogously to the IdA model, there exists a significant correlation between empirical and theoretical results. Additionally, it can be appreciated that in the case of the DiA model the slope is closer to 1 and, more importantly, consistent with that value according to the standard error. Hence, the DiA model improves the results of the IdA model and reproduces the correlations between A and R more faithfully.

7 Intermediate computations and fits

In this section we detail all the intermediate computations that need to be performed in order to obtain the efficiency distribution for each one of the three proposed models: the independent variables (InV) model, the identical actors (IdA) model and the distinguishable actors (DiA) model. In particular, we present fits of the data to heavy-tailed distributions performed with the powerlaw python package [27].

7.1 Independent variables model

In the InV model we fit the probability density functions (PDFs) of activity A and response R to two different statistical distributions: the power law (PL) and the exponentially truncated power law (TPL). The former is used to characterize the deviation of this model from the empirical results and the latter to reproduce the empirical distribution of efficiency. In this section, we present the resulting fits of the data to those models.

The expression employed for the power law distribution is the following:

$$f(x) = (\gamma - 1)x_{min}^{\gamma-1}x^{-\gamma} \tag{S19}$$

Where x_{min} corresponds to the minimum value of x. The exponentially truncated power law distribution can be written as:

$$f(x) = \frac{\lambda^{1-\alpha}}{\Gamma(1-\alpha, \lambda x_{min})}x^{-\alpha}e^{-\lambda x} \tag{S20}$$

7.1.1 Scientific citations

The efficiency distribution according to the InV model in the case of the scientific citations datasets was computed by adopting the truncated power law models for the $f_R(R)$ and $f_A(A)$ PDFs. However, as the empirical distributions present a very noisy behavior towards the tail, instead of using the whole datasets, the data selected to perform the fits corresponded to $A \in [1, 30]$ and $R \in [2, 300]$. The resulting fits are shown in green in figures S5 and S6, where the raw data are represented as blue dots and the data used to perform the fits as orange squares. Notice that, although the fits were not performed using the full range of the data, the distributions of efficiency shown in the main document were computed without filtering out any data. The parameters α and λ of Eq. (S20) computed for each fit are presented in table S3.

7.1.2 Twitter

The distributions of activity and response for the Twitter datasets were also modeled with TPL. Additionally, the distribution of response and the right tail of the distribution of efficiency for Twitter were also fit to a regular PL in order to determine the departure of the InV model with respect to the data. The fits that have been obtained are presented in figures S7, S8 and S9. The parameters of Eq. (S19) and Eq. (S20) for the response distributions as well as the parameters of Eq. (S20) for the activity distribution are presented in table S4.

7.1.3 Wikipedia

In the case of the Wikipedia dataset, the distributions of A and R have been modeled with TPL. The resulting fits are presented in figures S10 and S11 and the parameters of Eq. (S20) in table S5.

7.2 Identical actors model

The IdA model requires the distribution of response to single actions $p(r)$ and the distribution of activity $p(A)$ as InVuts. In the main document three different methodologies are proposed to solve this model: Monte-Carlo simulation, direct numerical computation (Eq. (10), Eq. (17) and Eq. (18) of the main document) and an analytical approximation (Eq. (11) of the main document). In the first two methodologies a hybrid approach (explained above) is adopted to fit the empirical distributions of r for some of the systems (for others, a regular fit is performed). Here, we present the resulting fits for every system.

In the hybrid methodology, the tail of the $p(r)$ distribution is modeled as a lognormal with the following expression:

$$f(x) = \sqrt{\frac{2}{\pi\sigma^2}} \left[\operatorname{erfc}\left(\frac{\log x_{min} - \mu}{\sqrt{2}\sigma} \right) \right] \frac{1}{x} e^{-\frac{(\log x - \mu)^2}{2\sigma^2}} \tag{S21}$$

With respect to the analytical approximation of the model (Eq. (11) of main document), both r and A are modeled as continuous random variables distributed as power laws. These fits are also presented in this section.

7.2.1 Scientific citations

The distribution of activity $p(A)$ of the scientific citations network for the numerical computation of the IdA model was modeled as a TPL and the resulting fit was already shown in figure S5. In order to compute the analytical approximation of the efficiency distribution, $p(A)$ was also fit to a power law. The resulting fits are shown in figure S12 and the corresponding parameters of Eq. (S19) in table S3.

In order to compute the IdA model numerically, The $p(r)$ on the other hand was fit to a lognormal distribution using the hybrid methodology for the interval $r \in [1, \infty)$ and maintaining the empirical probability for $r = 0$. The results are shown in figure S13 and the parameters of the fit are presented in table S3. For the analytical approximation of the IdA model, we have modeled $p(r)$ as a PL. We have fit the data of r to a PL by computing the optimal r_{min} that optimizes the Kolmogorov-Smirnov distance [28] with the methodology implemented in [27]. The results are shown in figure S14 and the parameters of the fits presented in table S3. As can be seen, whereas the hybrid distributions of figure S13 show a very good agreement with the data, the PL fits of figure S14 are only good for the tail. The lower values of r present a large divergence between the data and the fitting line. This causes the analytical approximation of the IdA model to perform poorly in the case of the scientific citations datasets, as will be discussed in section 7.2.4.

7.2.2 Twitter

The activity distribution of the Twitter datasets for the numerical computation of the IdA model was modeled as a TPL, so the resulting fit was already shown in figure S7. In order to compute the analytical approximation of the efficiency distribution, the $p(A)$ was also fit to a power law. The resulting fits are also shown in figure S7 and the corresponding parameters of Eq. (S19) in table S4.

The $p(r)$ on the other hand was fit using the hybrid methodology to a lognormal distribution for $r \in [1, \infty)$ maintaining the empirical probability for $r = 0$ in order to compute the IdA model numerically. The results are shown in figure S15 and the parameters of the fit are presented in table S4. For the analytical approximation of the IdA model, we have modeled $p(r)$ as a PL. We have fit the data of r to a PL following the same methodology as for the scientific citations datasets. The results are shown in figure S16 and the parameters of the fits presented in table S4.

7.2.3 Wikipedia

The distribution of activity in the case of Wikipedia was fit to a TPL to compute the IdA model. The result was shown in figure S10. The analytical approximation of the model was computed with a PL fit of the activity distribution, which is also presented in figure S10. The parameters of Eq. (S19) can be consulted in table S5.

The $p(r)$ on the other hand was fit using the hybrid methodology to a PL distribution for $r \in [4, \infty)$ maintaining the empirical probability for $r \in [0, 4)$ in order to compute the IdA model numerically. The results are shown in figure S17 and the parameters of the fit are presented in table S5. For the analytical approximation of the IdA model, we have modeled $p(r)$ as a PL. We have fit the data of r to a PL following the same methodology as for the scientific citations and Twitter datasets. The results are shown in figure S18 and the parameters of the fits presented in table S5.

7.2.4 The error associated to the PL fit of $p(r)$ affects the quality of the IdA model's analytical approximation

In the main document it is observed that the analytical approximation of the IdA model shows different levels of agreement with the Monte-Carlo simulations and the numerical computations. Here we show that the main cause for this deviation is the error associated to the power law fit of the distribution of response to single actions $p(r)$.

We have focused on the differences in the right tail of the efficiency distribution between the numerical and analytical computations. Given the heterogeneous nature of this distribution, we have measured these differences as the mean squared error of the logarithm of the probabilities. Therefore, if $f_{num}(\eta_i)$ is the average probability density associated to interval i of the histogram according to the numerical computation

and $f_{appr}(\eta_i)$ is the average probability density associated to the same interval according to the analytical derivation, the mean squared error ϵ_η would be:

$$\epsilon_\eta = \sqrt{\frac{\sum_{i=1}^{n}(\log[f_{appr}(\eta_i)] - \log[f_{num}(\eta_i)])^2}{n}} \tag{S22}$$

Where n is the number of bins used to compute the histogram. We have measured the error committed when $p(r)$ is fit to a power law in an analogous way. Let p_r^{emp} the empirical probability associated to value r of the response to single actions and p_r^{fit} the probability associated to the same value according to the power law fit. The mean squared error ϵ_r associated to this fit would be:

$$\epsilon_r = \sqrt{\frac{\sum_r(\log[p_r^{fit}] - \log[p_r^{emp}])^2}{n}} \tag{S23}$$

In this case the sum runs over all the possible values of r with non-zero empirical probabilities.

In figure S19 the relationship between ϵ_r and ϵ_η can be clearly appreciated. It is worth pointing out that this relationship seems to follow the same tendency for all the systems under study. Figure S19 also shows that the scientific citations system presents the worse agreements between the analytical approximation and numerical computation of the IdA model due to the lower quality of the PL fits of the $p(r)$ distribution.

7.3 Distinguishable actors model

The DiA model is defined by the conditional distribution of response for single actions r given the feature vector $\vec{s}$ of the individual: $p(r|\vec{s})$. Twitter was the only system analyzed under this model because of the inherent complication of defining and quantifying the relevant characteristics of an individual that determine her ability to trigger a response in an arbitrary setting. In the case of Twitter, the response only depends on the number of followers F of the individual and on an infection probability p_{inf} that is considered constant for every action and every individual:

$$p(r|\vec{s}) = p(r|F) = B(r; F, p_{inf}) \tag{S24}$$

Where $B(k; n, p)$ is a binomial distribution of a variable k with n Bernoulli trials and p success probability. The distribution of efficiency is computed in this case with Eq. (16), Eq. (17) and Eq. (18) of the main document.

Consequently, in order to solve the DiA model for Twitter it is necessary to compute the value of p_{inf}, which is accomplished by performing a Maximum Likelihood Estimation (MLE) of $p(r)$, and fit $p(F)$, which is carried out with the hybrid methodology presented in the Materials and Methods section of the main document. In this section we present the results for the MLE estimation of p_{inf} and the fits of the follower distribution $p(F)$.

The effective p_{inf} of a given dataset can be computed by maximizing the following log-likelihood function:

$$\mathcal{L} = \sum_i \log\left[\sum_F p(F)B(r_i^{obs}; F, p_{inf})\right] \tag{S25}$$

Where r_i^{obs} are observed values of response to single actions. However, given the enormous quantity of data, the optimization process can be very slow. In order to perform the computations faster, samples of the r data for each dataset were employed instead of the whole dataset. Specifically, we took 10 different samples of 1000 r_i^{obs} each and computed the average p_{inf} for each dataset, obtaining also a standard deviation. The results are shown in table S6.

The hybrid distribution of $p(F)$, which has been obtained by modeling the tail of the distribution with a PL, is shown in figure S20 and the corresponding parameters in table S4.

Table S3: Parameters of each fit performed with the scientific citations datasets.

**Dataset**	A			R			η	r				
	**TPL**		**PL**	**TPL**		**PL**	**PL**	**Hybrid lognormal**			**PL**	
	α	λ	γ	α	λ	γ	γ	q	μ	σ	x_{min}	γ
Argentina	2,273	8,237E-02	2,745	1,106	1,174E-02	1,564	1,696	1	2,085	1,174	31	2,676
Brazil	1,777	1,004E-01	2,442	1,443	5,039E-03	1,663	1,824	1	1,905	1,168	37	2,896
Chile	2,058	8,609E-02	2,592	1,062	9,894E-03	1,524	1,696	1	2,087	1,282	46	2,732
Colombia	2,324	2,839E-02	2,540	1,000	6,063E-03	1,438	1,702	1	1,851	1,373	29	2,541
Cuba	2,757	4,929E-03	2,793	1,000	5,689E-03	1,401	1,855	1	1,841	1,286	37	2,698
Finland	1,890	7,885E-02	2,438	1,050	1,388E-02	1,560	1,723	1	2,486	1,184	63	2,679
Greece	2,134	6,302E-02	2,554	1,127	1,046E-02	1,560	1,841	1	2,249	1,219	39	2,705
Iceland	2,239	1,058E-01	2,808	1,000	8,178E-03	1,413	1,489	1	2,566	1,363	26	2,130
Israel	2,192	5,861E-02	2,581	1,137	1,152E-02	1,576	1,733	1	2,319	1,271	95	2,760
New Zealand	2,121	5,215E-02	2,494	1,121	9,706E-03	1,548	1,685	1	2,341	1,199	55	2,693
Poland	1,694	8,122E-02	2,310	1,250	6,862E-03	1,577	1,835	1	1,840	1,241	30	2,617
Portugal	2,055	5,369E-02	2,450	1,140	1,053E-02	1,567	1,745	1	2,324	1,181	51	2,890
Spain	1,687	1,056E-01	2,401	1,332	9,494E-03	1,657	1,911	1	2,349	1,211	87	2,837
Venezuela	1,008	4,269E-01	2,873	1,183	1,156E-02	1,599	1,585	1	1,679	1,364	38	2,542

Table S4: Parameters of each fit performed with the Twitter datasets.

**Dataset**	A			R			η	r					F		
	**TPL**		**PL**	**TPL**		**PL**	**PL**	**Hybrid lognormal**			**PL**		**Hybrid TPL**		
	α	λ	γ	α	λ	γ	γ	q	μ	σ	x_{min}	γ	q	α	λ
Spanish elections 2016	1,864	5,407E-03	1,935	1,471	5,478E-04	1,535	1,937	1	-0,136	1,889	125	2,509	500	1,806	1,067E-08
Spanish elections 2015	1,903	3,891E-03	1,956	1,476	7,432E-04	1,547	1,965	1	-0,193	1,846	60	2,465	500	1,813	1,100E-08
Dock workers conflict	2,066	2,352E-03	2,092	1,477	4,368E-04	1,535	1,736	1	-1,665	2,607	41	2,119	500	1,625	3,462E-07
Palestine	2,171	1,614E-03	2,187	1,576	2,086E-04	1,605	1,725	1	-3,197	2,864	41	2,101	500	1,610	9,378E-08
PSOE crisis	1,597	5,649E-03	1,719	1,470	2,550E-04	1,519	1,850	1	-1,877	2,434	222	2,549	500	1,817	1,368E-07
Catalan elections 2015	2,107	1,009E-02	2,179	1,387	2,506E-03	1,526	1,854	1	0,035	1,806	10	2,138	500	1,789	5,998E-07
Basque elections 2016	2,302	9,530E-07	2,302	1,405	1,544E-03	1,518	1,767	1	0,124	1,865	11	2,193	500	1,631	5,265E-07
Regional elections 2015	2,119	4,747E-03	2,160	1,418	2,923E-03	1,555	1,942	1	0,041	1,622	7	2,225	500	1,768	6,663E-07
Madrid-Barcelona match	2,408	5,166E-03	2,436	1,751	8,344E-06	1,753	1,723	1	-990,155	37,169	2	1,706	500	1,729	7,164E-09
HSR conflict	1,988	4,346E-04	1,997	1,338	6,188E-04	1,441	1,746	1	1,290	1,424	36	2,575	500	1,611	6,389E-08
PSOE primary	2,438	3,240E-03	2,456	1,563	1,001E-03	1,624	1,782	1	-1,084	2,234	32	2,251	500	1,605	3,774E-07
FARC referendum	2,045	8,271E-03	2,113	1,485	9,829E-04	1,562	1,795	1	-1,268	2,228	7	1,981	500	1,561	1,496E-07
Turkish referendum	2,481	7,584E-03	2,516	1,559	5,781E-04	1,607	1,747	1	-1,516	2,412	90	2,712	500	1,518	2,928E-08
Argentinian retirement plans	1,825	3,261E-03	1,881	1,553	7,963E-05	1,574	1,835	1	-4,597	3,220	4	1,791	500	1,772	2,955E-09

Table S5: Parameters of each fit performed with the Wikipedia data.

A			R			η	r				
**TPL**		**PL**	**TPL**		**PL**	**PL**	**Hybrid lognormal**			**PL**	
α	λ	γ	α	λ	γ	γ	q	μ	σ	x_{min}	γ
1,521	1,222E-04	1,543	1,848	4,790E-04	1,864	2,784	4	-0,606	0,938	4	3,966

Table S6: Values of p_{inf} estimated through MLE for the Twitter datasets. The averages and standard deviations obtained for 10 samples of 1000 instances each are presented.

**Dataset**	$\bar{p}_{inf}/10^{-3}$	$\Delta p_{inf}/10^{-3}$
Spanish elections 2016	1,56	0,14
Spanish elections 2015	1,41	0,08
Dock workers conflict	1,05	0,06
Palestine	0,47	0,04
PSOE crisis	1,07	0,10
Catalan elections 2015	3,2	0,4
Basque elections 2016	1,69	0,14
Regional elections 2015	1,64	0,09
Madrid-Barcelona match	0,23	0,04
HSR conflict	5,3	0,5
PSOE primary	0,50	0,05
FARC referendum	0,41	0,06
Turkish referendum	0,88	0,08
Argentinian retirement plans	0,69	0,07

References

[1] ElPaís. Spanish general election 2015: As it happened. https://elpais.com/elpais/2015/12/18/media/1450434499_422758.html, 2015. Online, accessed 10-July-2019.

[2] S Martin-Gutierrez, JC Losada, and RM Benito. Recurrent patterns of user behavior in different electoral campaigns: A twitter analysis of the spanish general elections of 2015 and 2016. *Complexity*, 2018, 2018.

[3] Sarah Rainsford. Spanish election: PP wins most seats but deadlock remains. https://www.bbc.com/news/world-europe-36632276, 2016. Online, accessed 10-July-2019.

[4] Spanish ports stevedoring conflict. https://www.uecc.com/news/2017/may/spanish-ports-stevedoring-conflict/, 2017. Online, accessed 4-April-2019.

[5] Sam Jones. Pedro Sánchez insists he is still in charge of Spanish Socialist party. https://www.theguardian.com/world/2016/sep/29/pedro-sanchez-insists-still-charge-spanish-socialist-party, 2017. Online, accessed 10-July-2019.

[6] ElPaís. Catalan elections 2015: As they happened. https://elpais.com/elpais/2015/09/27/inenglish/1443344688_042394.html, 2015. Online, accessed 10-July-2019.

[7] 2016 Basque regional election. https://en.wikipedia.org/wiki/2016_Basque_regional_election, Mar 2019. Online, accessed 4-April-2019.

[8] 2015 Spanish regional elections. https://en.wikipedia.org/wiki/2015_Spanish_regional_elections, Jan 2019. Online, accessed 4-April-2019.

[9] Partido Real Madrid - FC Barcelona en directo. https://www.laliga.es/directo/temporada-2017-2018/laliga-santander/17/real-madrid_barcelona, 2017. Online, accessed 10-December-2018.

[10] Wikipedia. Anexo: Clubes españoles de fútbol ganadores de competiciones nacionales e internacionales — Wikipedia, the free encyclopedia, 2018. Online, accessed 10-December-2018.

[11] High-speed rail tunnel protests spread to the centre of the city of Murcia. https://murciatoday.com/high_speed-rail-tunnel-protests-spread-to-the-centre-of-the-city-of-murcia_124400-a.html, 2017. Online, accessed 4-April-2019.

[12] 2017 Spanish Socialist Workers' Party leadership election. https://en.wikipedia.org/wiki/2017_Spanish_Socialist_Workers'_Party_leadership_election, Dec 2018. Online, accessed 4-April-2019.

[13] Colombia referendum: Voters reject Farc peace deal. https://www.bbc.com/news/world-latin-america-37537252, Oct 2016. Online, accessed 27-June-2019.

[14] Burhan Yüksekkaş and Raf Sanchez. Erdogan claims victory in turkish referendum but result swiftly challenged by opposition. https://www.telegraph.co.uk/news/2017/04/16/erdogan-claims-victory-turkish-referendum-result-swiftly-challenged/, Apr 2017. Online, accessed 28-June-2019.

[15] Luc Cohen. Argentina Congress passes pension reform after protests, clashes. https://www.reuters.com/article/us-argentina-pensions/argentina-congress-passes-pension-reform-after-protests-clashes-idUSKBN1ED18S, Dec 2017. Online, accessed 4-April-2019.

[16] Web of science. https://clarivate.com/products/web-of-science/. Last accessed: 3-4-2019.

[17] Stanford large network dataset collection. https://snap.stanford.edu/data/. Last accessed: 3-4-2019.

[18] J. H. Curtiss. On the distribution of the quotient of two chance variables. *The Annals of Mathematical Statistics*, 12(4):409–421, 1941.

[19] M. D. Springer. *The algebra of random variables*. J. Wiley & Sons, 1979.

[20] David Kempe, Jon Kleinberg, and Éva Tardos. Maximizing the spread of influence through a social network. In *Proceedings of the ninth ACM SIGKDD international conference on Knowledge discovery and data mining*, pages 137–146. ACM, 2003.

[21] Alfredo J Morales, Javier Borondo, Juan Carlos Losada, and Rosa M Benito. Efficiency of human activity on information spreading on twitter. *Social Networks*, 39:1–11, 2014.

[22] David R Bild, Yue Liu, Robert P Dick, Z Morley Mao, and Dan S Wallach. Aggregate characterization of user behavior in twitter and analysis of the retweet graph. *ACM Transactions on Internet Technology (TOIT)*, 15(1):4, 2015.

[23] Javier Borondo, AJ Morales, Juan Carlos Losada, and Rosa M Benito. Characterizing and modeling an electoral campaign in the context of twitter: 2011 spanish presidential election as a case study. *Chaos: an interdisciplinary journal of nonlinear science*, 22(2):023138, 2012.

[24] Hai-Bo Hu and Xiao-Fan Wang. Disassortative mixing in online social networks. *EPL (Europhysics Letters)*, 86(1):18003, apr 2009.

[25] Haewoon Kwak, Changhyun Lee, Hosung Park, and Sue Moon. What is twitter, a social network or a news media? In *Proceedings of the 19th international conference on World wide web*, pages 591–600. AcM, 2010.

[26] Eytan Bakshy, Jake M Hofman, Winter A Mason, and Duncan J Watts. Everyone's an influencer: quantifying influence on twitter. In *Proceedings of the fourth ACM international conference on Web search and data mining*, pages 65–74. ACM, 2011.

[27] Jeff Alstott, Ed Bullmore, and Dietmar Plenz. powerlaw: A python package for analysis of heavy-tailed distributions. *PLOS ONE*, 9(1):1–11, 01 2014.

[28] Aaron Clauset, Cosma Rohilla Shalizi, and M. E. J. Newman. Power-law distributions in empirical data. *SIAM Review*, 51(4):661–703, 2009.

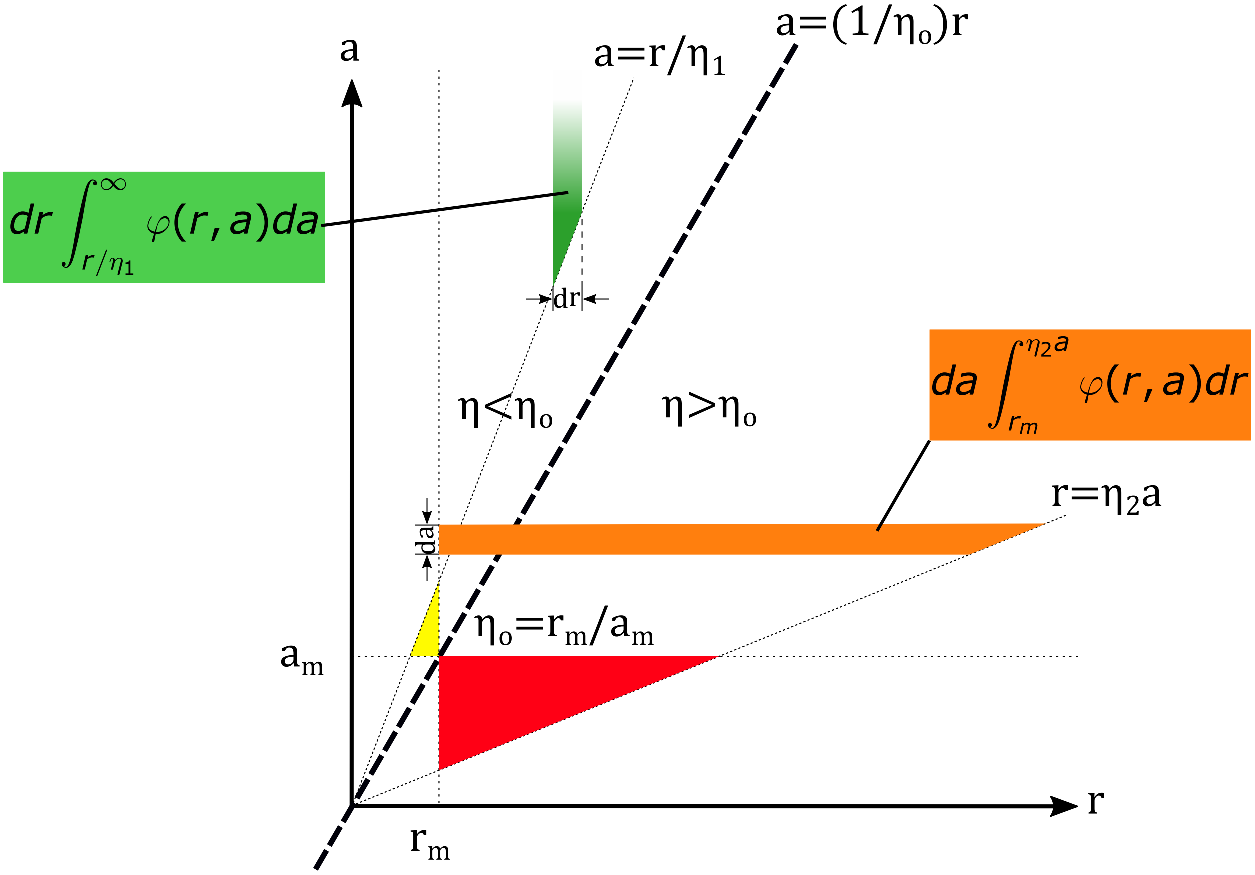

Figure S1: Diagram showing the regions of integration defined to each side of the line $\eta_o = \frac{r_m}{a_m}$ to obtain the cumulative distribution function of the efficiency.

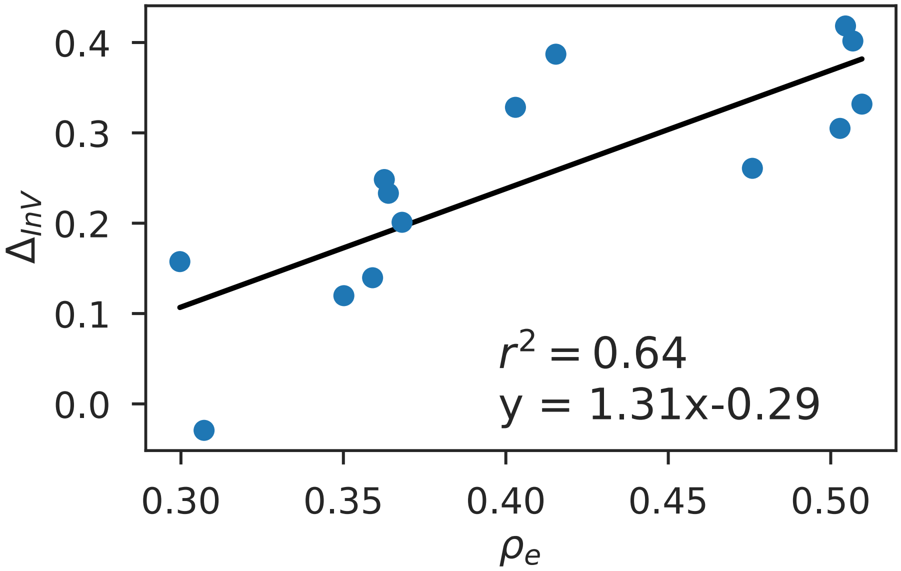

Figure S2: The deviation of the InV model (Δ_{InV}) with respect to the Twitter empirical data is related to the correlations between A and R, measured with the Spearman's correlation (ρ_e). The figure shows a monotonous increment of Δ_{InV} with ρ_e, which has been characterized by means of a linear regression.

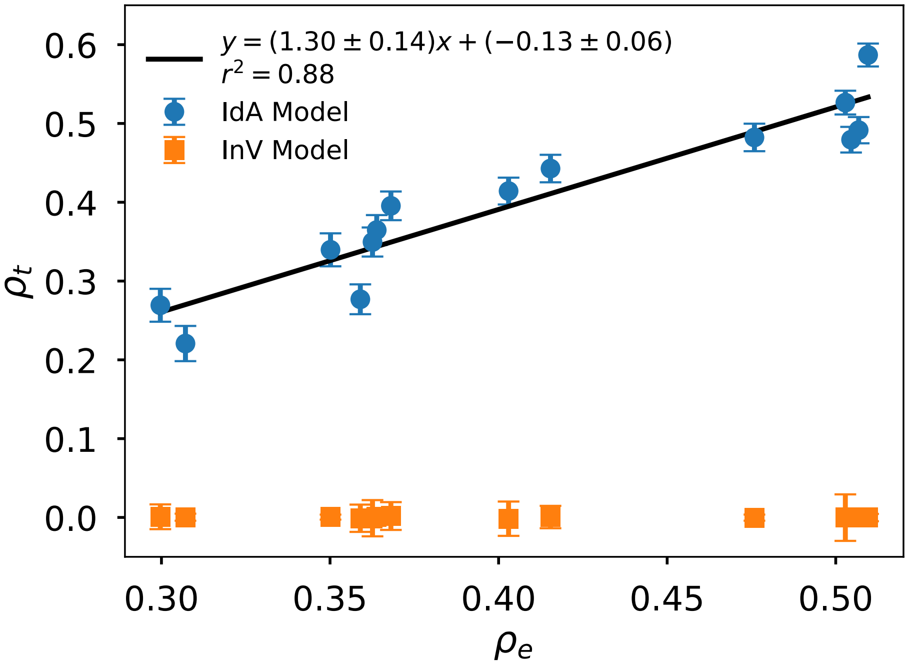

Figure S3: Linear regression of the correlation induced by the IdA model (blue dots) with respect to the empirical correlation computed for the Twitter datasets. Each point corresponds to a different conversation. For comparison the values corresponding to the InV model have also been plotted (orange squares).

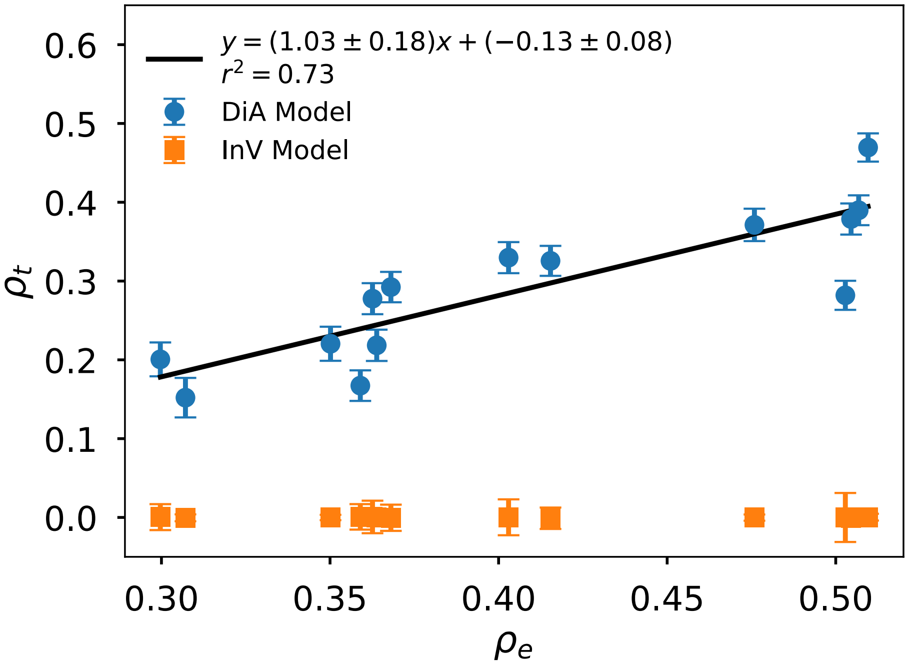

Figure S4: Linear regression of the correlation induced by the DiA model with respect to the empirical correlation computed for the Twitter datasets. Each point corresponds to a different conversation. The reference values obtained for the InV model are also displayed.

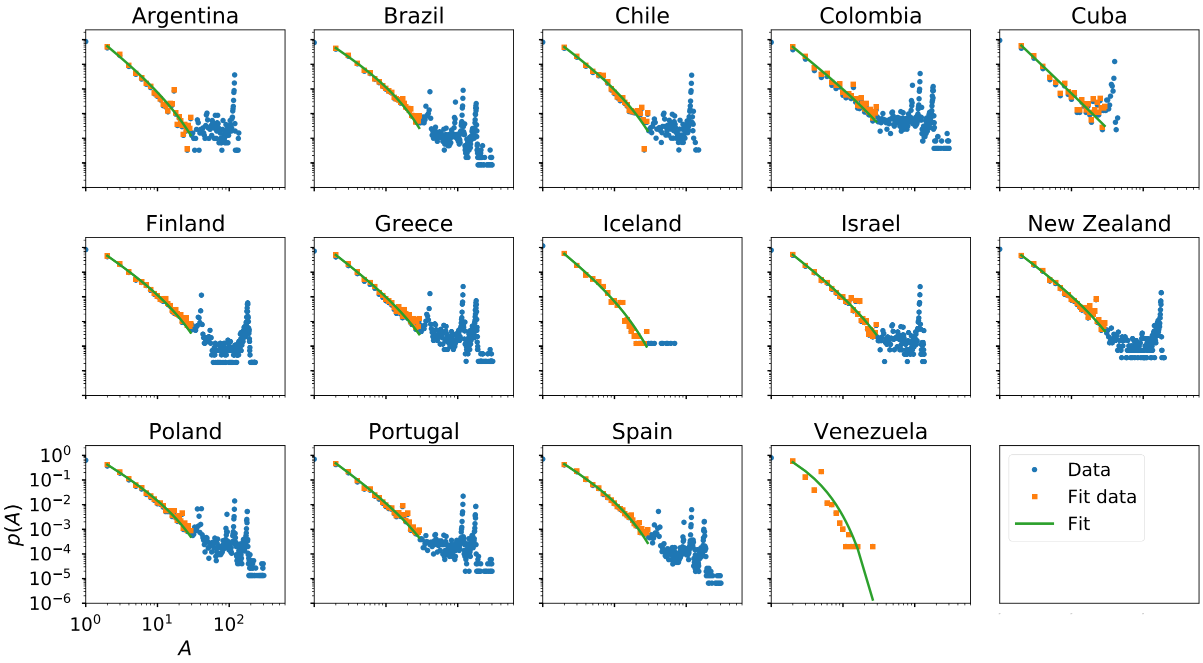

Figure S5: Fit of the empirical distribution of activity of the scientific citations network to a truncated power law. Blue dots correspond to the full data and orange squares to the data selected to perform the fit ($A \in [1, 30]$).

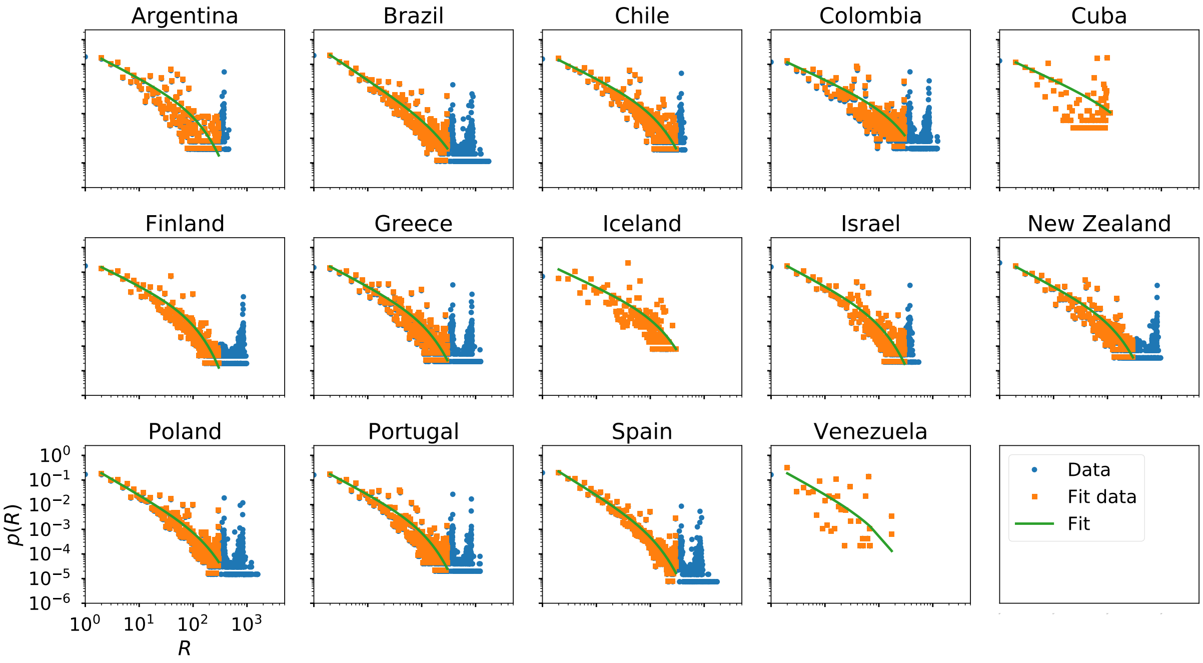

Figure S6: Fit of the empirical distribution of response of the scientific citations network to a truncated power law. Blue dots correspond to the full data and orange squares to the data selected to perform the fit ($R \in [2, 300]$).

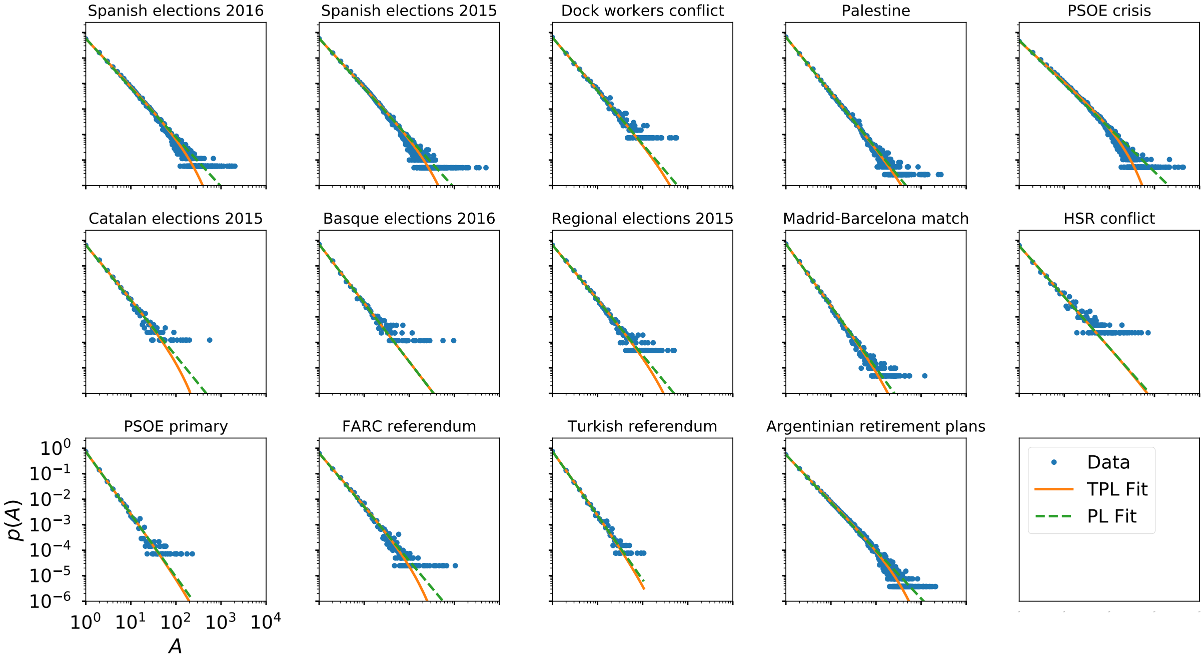

Figure S7: Fit of the empirical distribution of activity of the Twitter datasets to a power law (dashed green line) and to a truncated power law (continuous orange line).

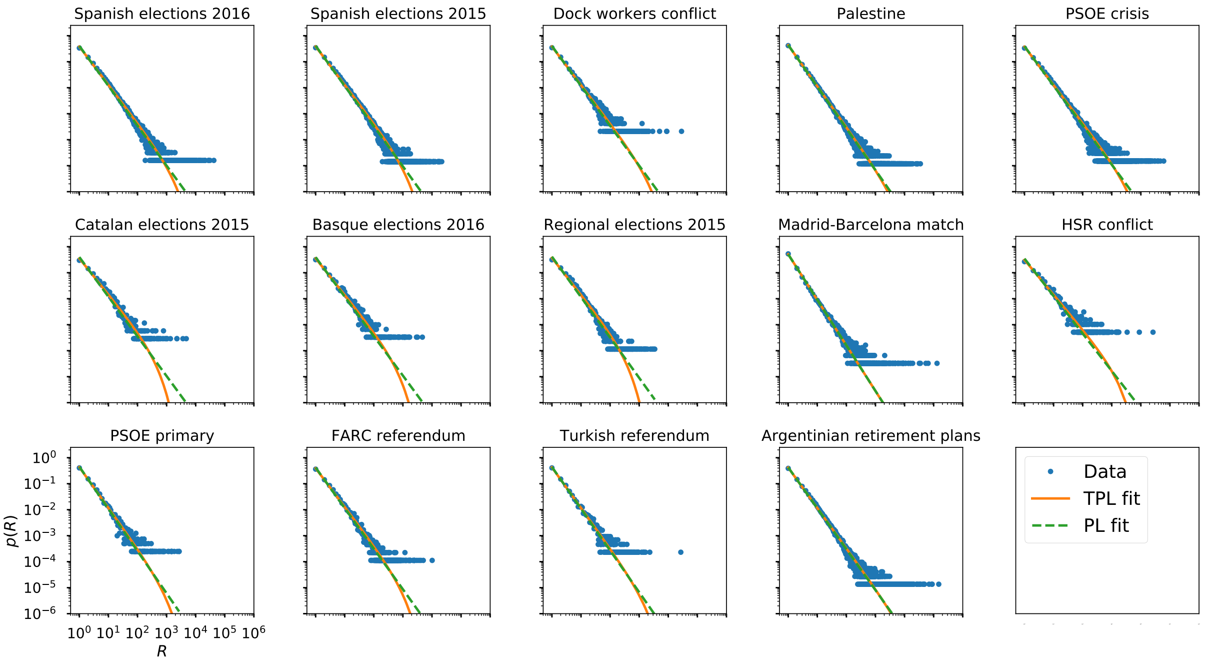

Figure S8: Fit of the empirical distribution of response of the Twitter datasets to a power law (dashed green line) and to a truncated power law (continuous orange line).

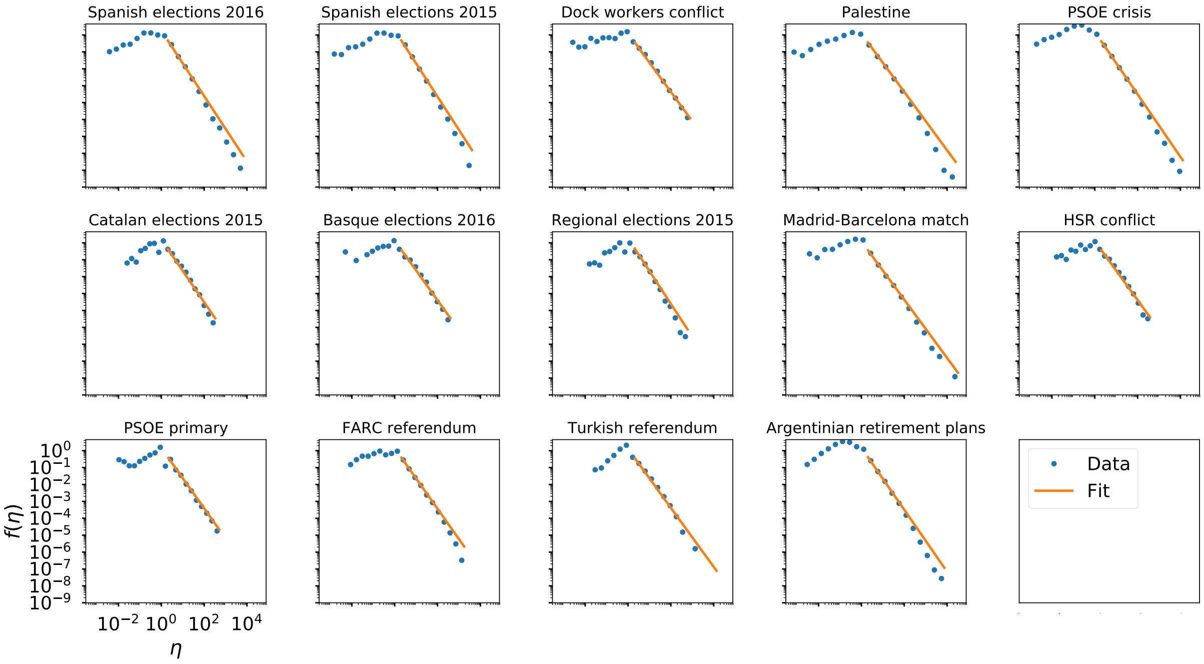

Figure S9: Fit of the right tail of the distribution of efficiency of the Twitter datasets to a power law.

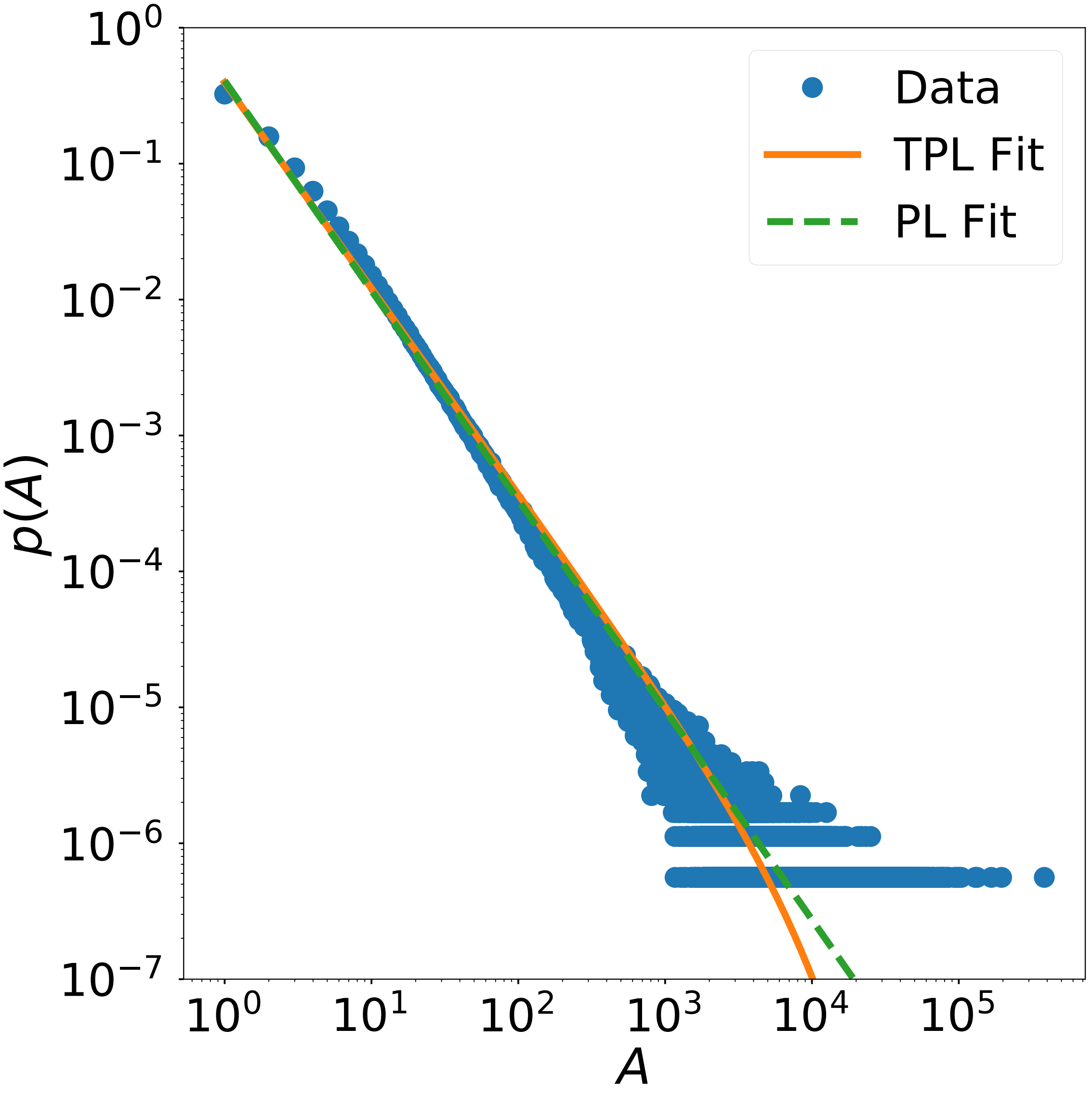

Figure S10: Fit of the empirical distribution of activity of the Wikipedia dataset to a power law (dashed green line) and to a truncated power law (continuous orange line).

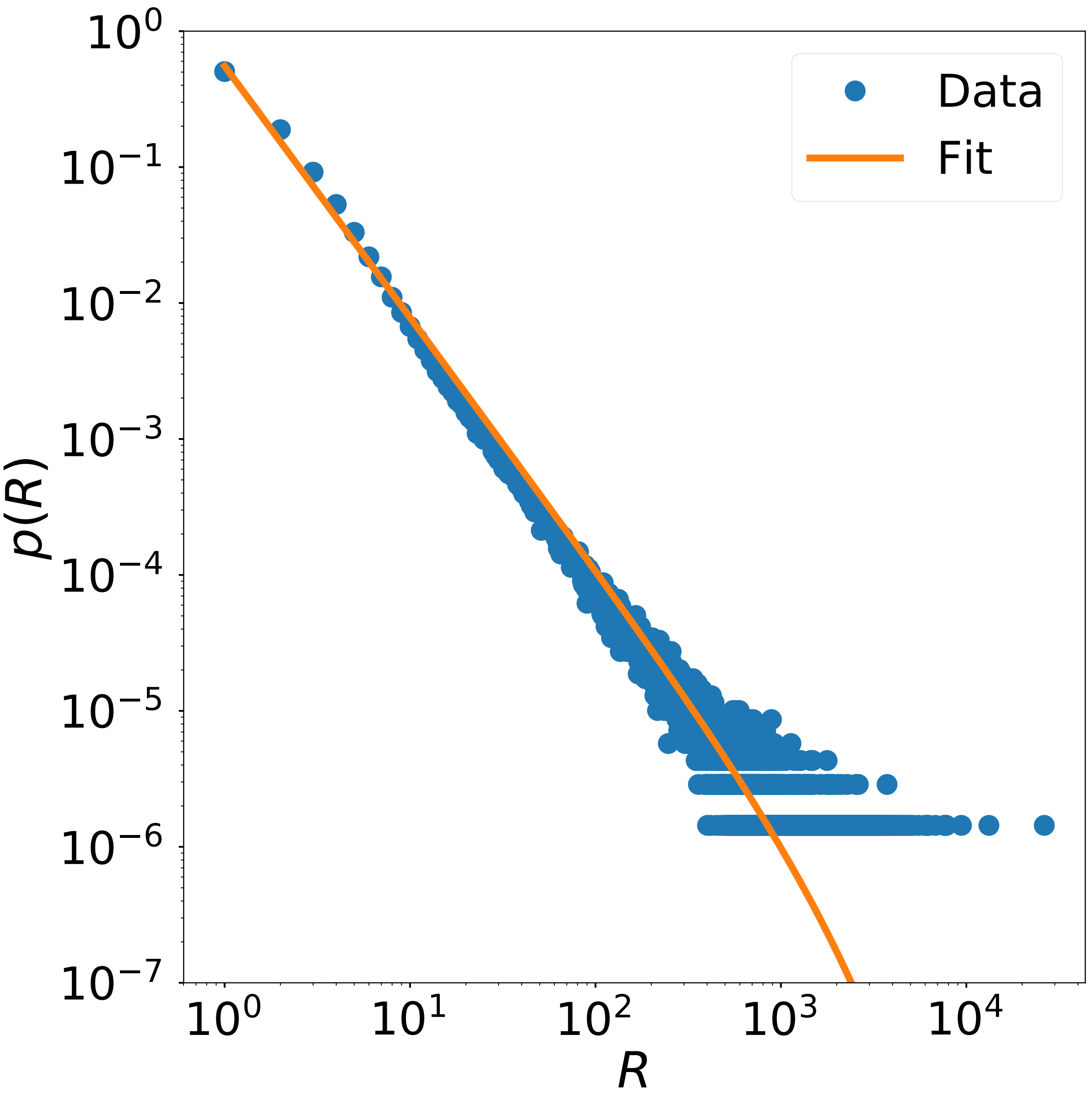

Figure S11: Fit of the empirical distribution of response of the Wikipedia dataset to a TPL.

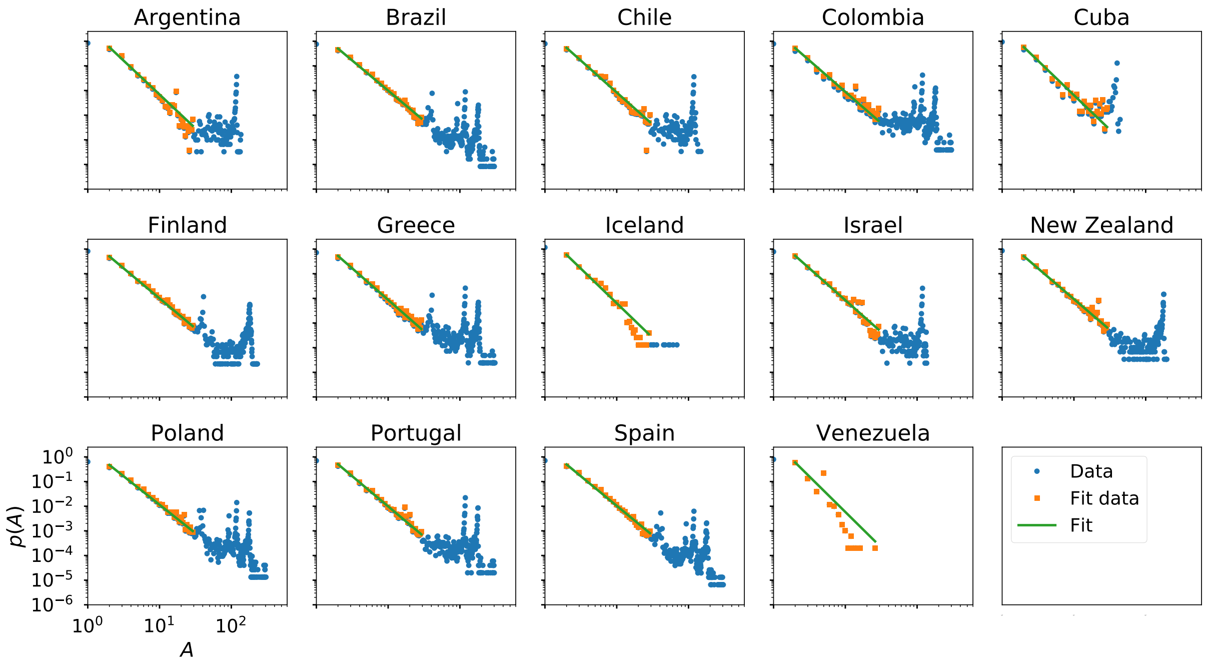

Figure S12: Fit of the empirical distribution of activity of the scientific citations network datasets to a PL.

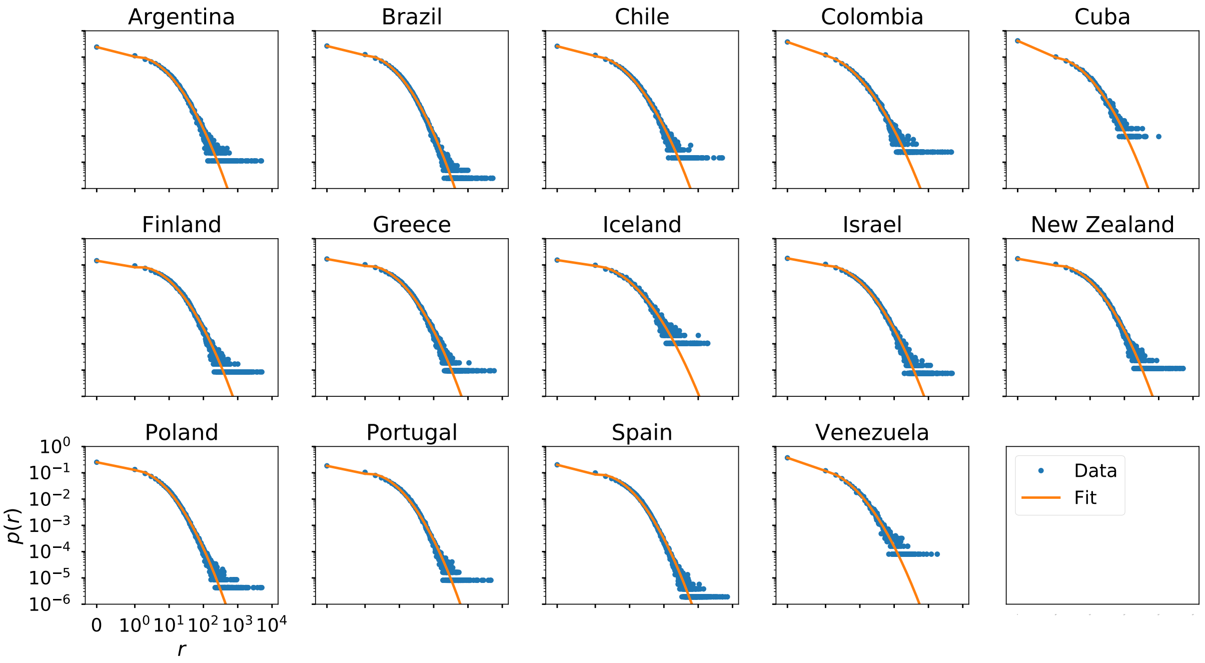

Figure S13: Hybrid lognormal fit of the empirical distribution of response to single actions of the scientific citations network datasets.

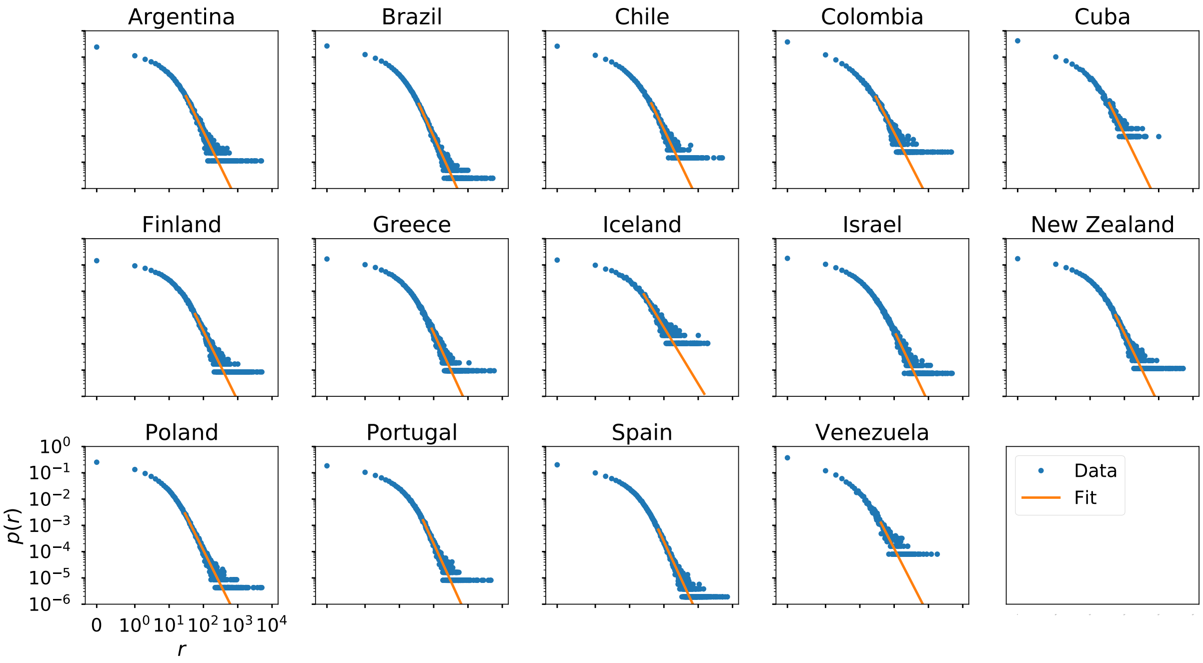

Figure S14: Power law fit of the empirical distribution of response to single actions of the scientific citations network datasets.

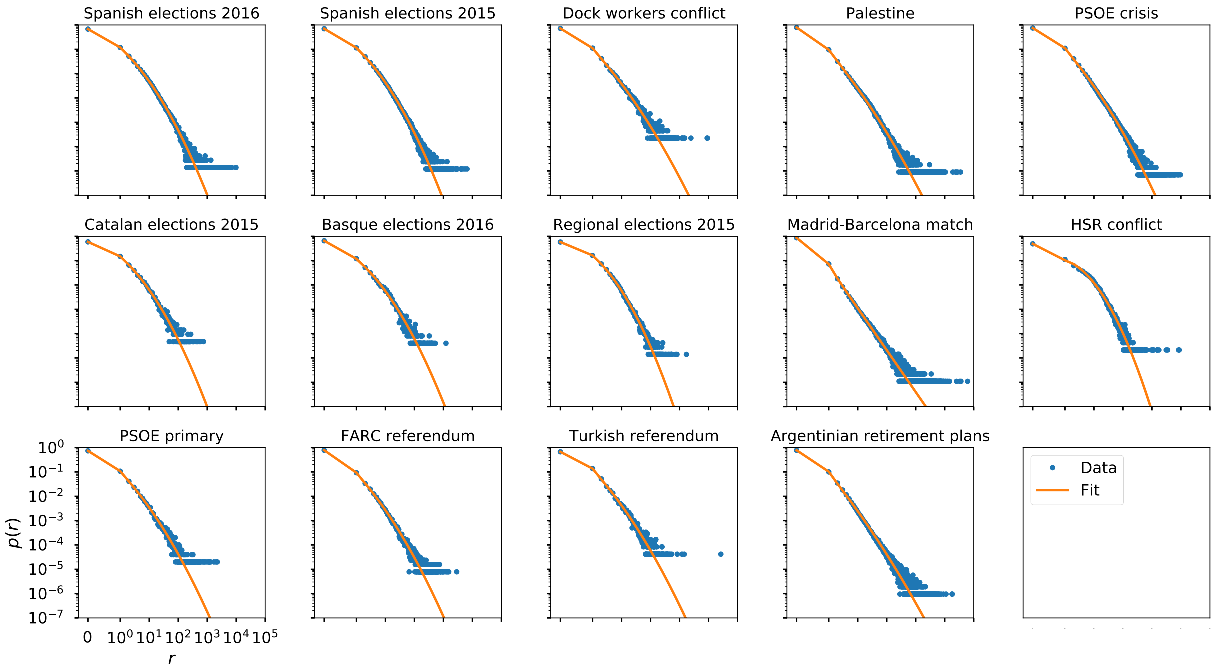

Figure S15: Hybrid lognormal fit of the empirical distribution of response to single actions of the Twitter datasets.

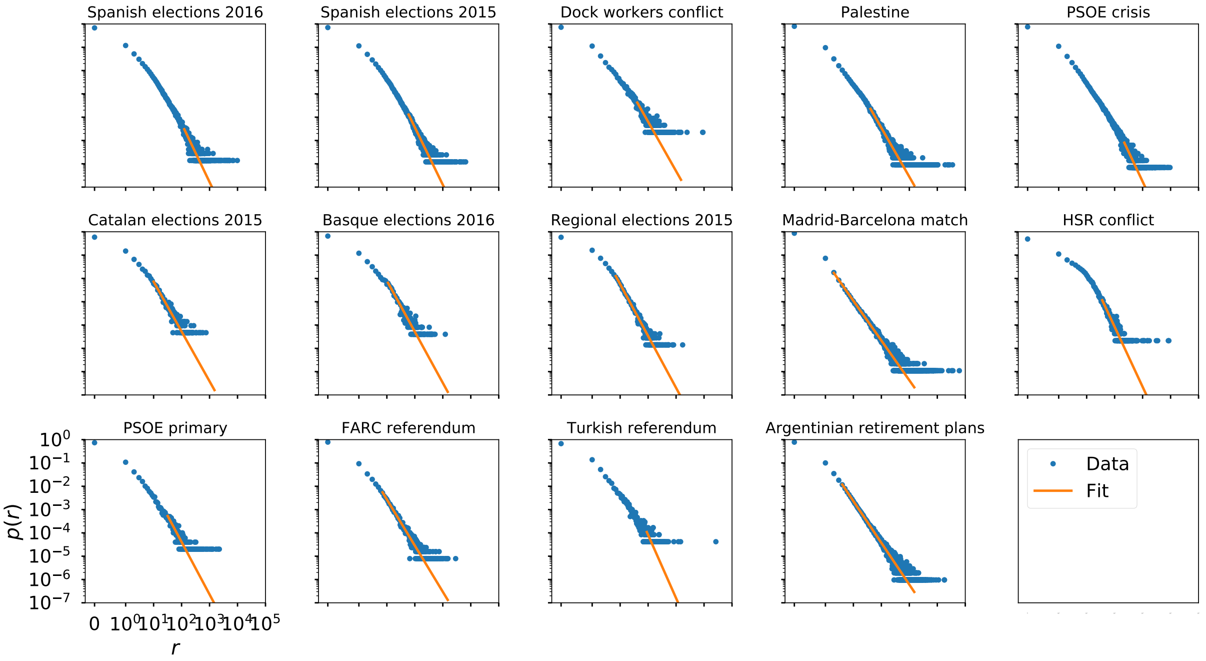

Figure S16: Power law fit of the empirical distribution of response to single actions of the Twitter datasets.

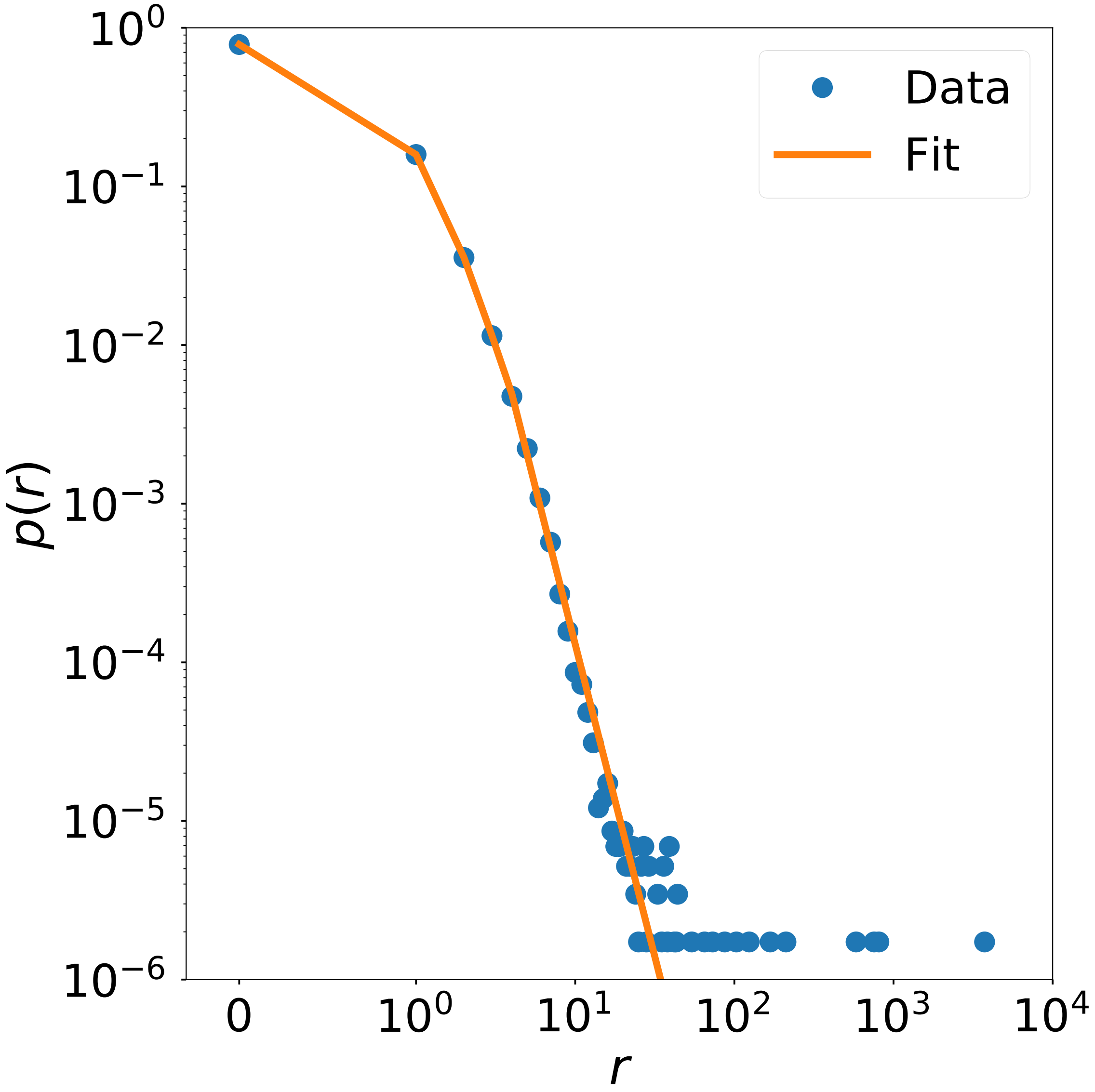

Figure S17: Hybrid PL fit of the empirical distribution of response to single actions of the Wikipedia dataset.

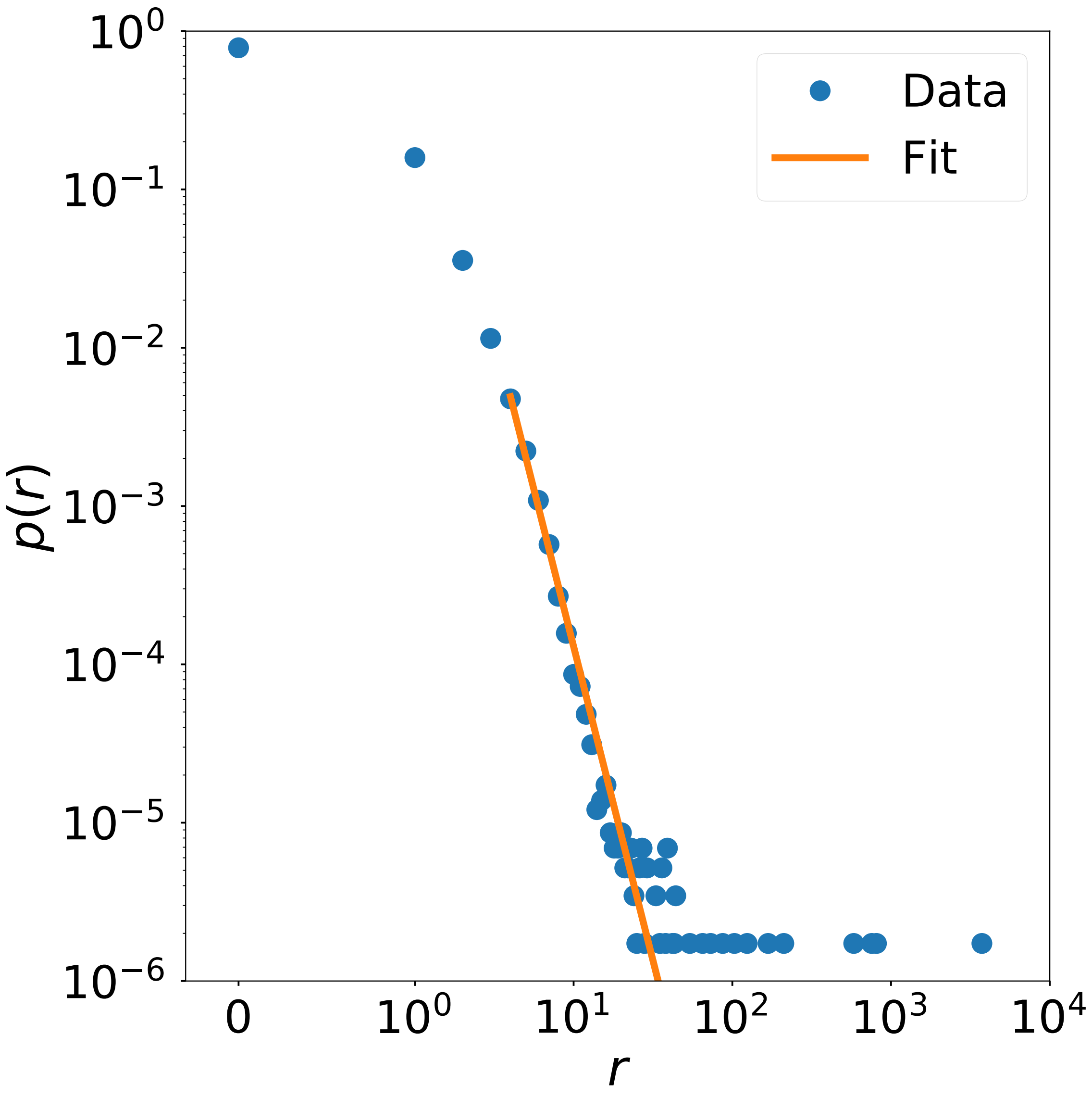

Figure S18: Power law fit of the empirical distribution of response to single actions of the Wikipedia dataset.

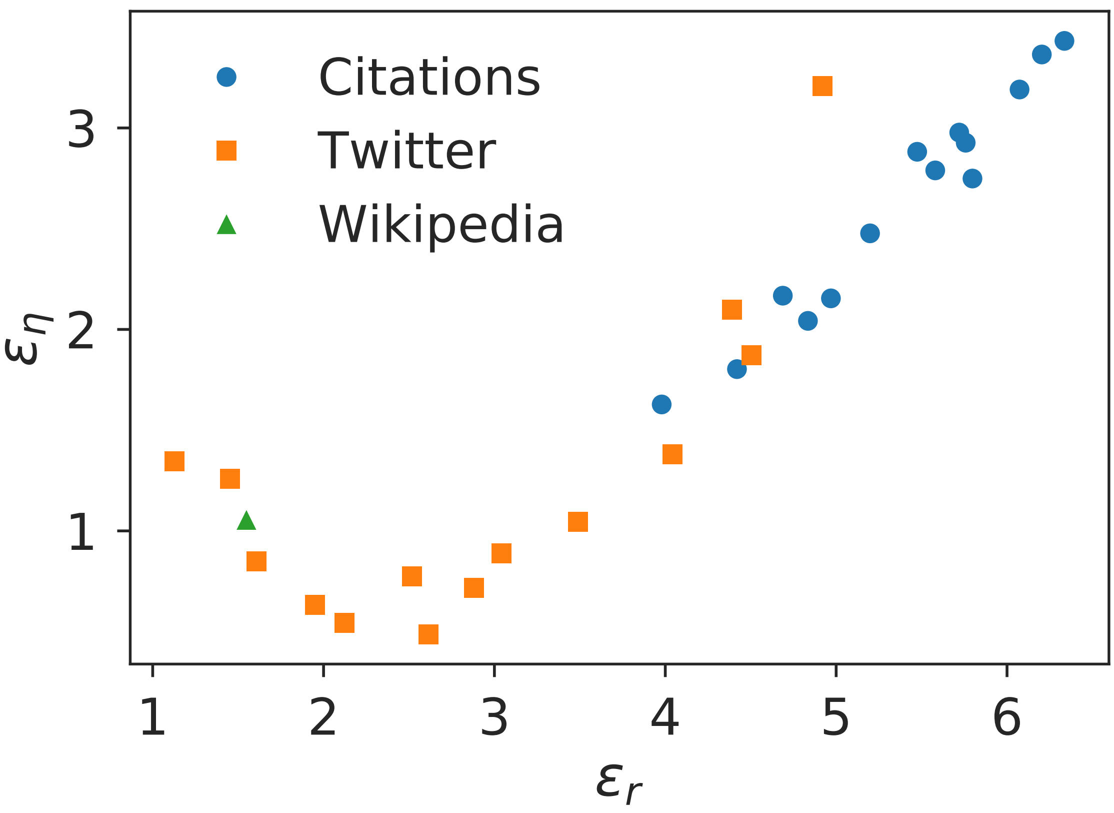

Figure S19: Relationship between the fitting error committed when $p(r)$ is modeled as a power law (ϵ_r) and the deviation of the analytical approximation with respect to the numerical computation of the IdA model (ϵ_η) in the right tail of the efficiency distribution $f(\eta)$.

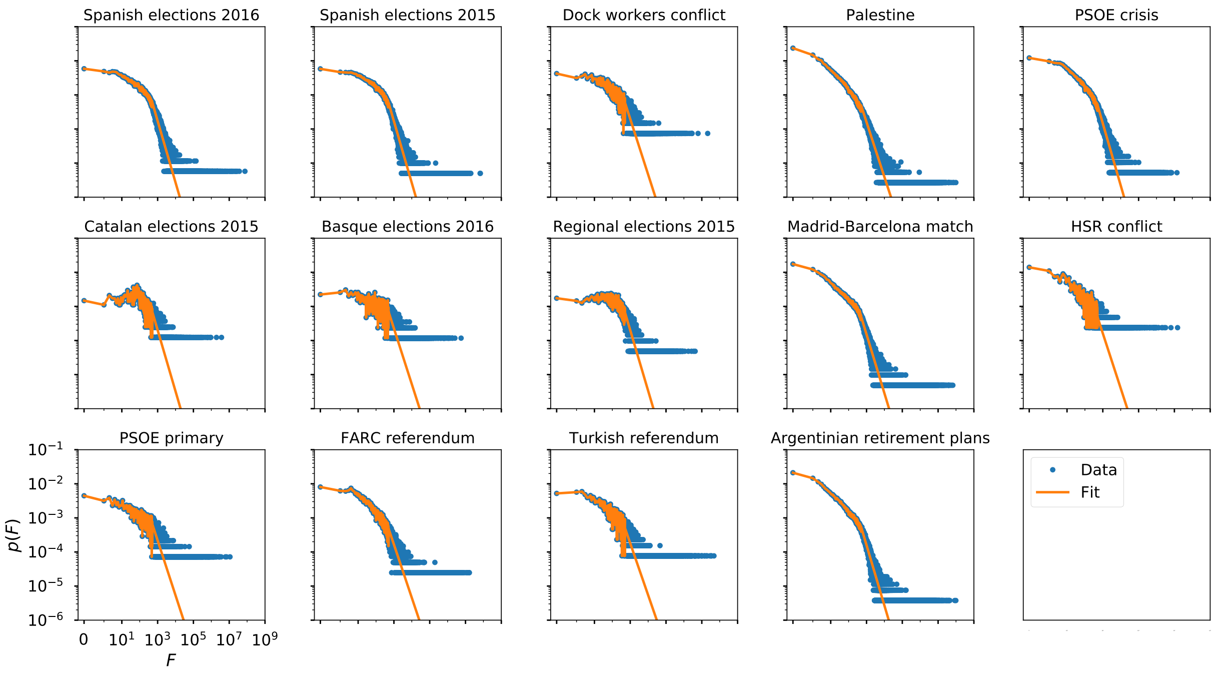

Figure S20: Fit of the empirical distribution of followers of the Twitter datasets to the empirical values with a PL tail.
